# The newly developed genomic-SSR markers uncover the genetic characteristics and relationships of olive accessions

**DOI:** 10.7717/peerj.8573

**Published:** 2020-02-13

**Authors:** Danyang Li, Cui Long, Xiaoming Pang, Delu Ning, Tao Wu, Mingliang Dong, Xiaoning Han, Huihong Guo

**Affiliations:** 1 National Engineering Laboratory for Tree Breeding, College of Biological Sciences and Technology, Beijing Forestry University, Beijing, China; 2 Yunnan Academy of Forestry and Grassland, Kunming, Yunnan, China

**Keywords:** Olive, Trinucleotide genomic-SSR, Genetic characteristics, Genetic relationships

## Abstract

**Background:**

Olive (*Olea europaea* L.) is an important oil and fruit crop worldwide, owning a rich germplasm with a large number of cultivars. Simple sequence repeats (SSRs) are excellent markers and have been used for the identification of olive cultivars. However, the limited number of SSR markers and the occurrence of confusion on the names of cultivars, as well as the possible appearance of clonal variation make it difficult to identify cultivars and interpret relationships among olive cultivars.

**Method:**

SSR markers were designed based on trinucleotide repeat sequences by screening the whole genome of olive, and the polymorphic SSR markers were developed that were applied to the identification of 53 olive accessions. The genetic characteristics and relationships of these olive accessions were evaluated based on the developed SSR markers.

**Results:**

Twenty-one highly polymorphic genomic-SSR markers were developed, covering most chromosomes of olive. These SSR markers could well distinguish all 53 olive accessions, confirming their effectiveness. DNA fingerprints of the 53 olive accessions were constructed based on the 21 SSR markers. The dendrogram clearly divided the tested accessions into two main groups, which was also supported by the results of principal coordinate analysis. A total of 31 private alleles were detected in 15 olive accessions, which reflected the genetic diversity within 53 olive accessions to some extent. Six homonymy cases were also clarified by genetic analysis. These results suggest that the newly developed olive SSR markers are informative for the exploitation, preservation and breeding of olive.

## Introduction

Olive (*Olea europaea* L.) is an important tree used as a source of food and oil in the world, which is praised as a “precious fruit” (Díez et al., 2015). The origin of olive can be traced to the eastern Mediterranean Coast, and the expansion of the Roman Empire favored the spread of olive all around the Mediterranean basin (Vossen, 2007), where accounts for more than 90% of the world’s olive oil production (Aksehirli-Pakyurek et al., 2017). During the recent decades, the olive has been spread to other areas for cultivation, such as the USA, South American, Australia, and China (Koubouris et al., 2019; Sion et al., 2019). Since 1964, many olive cultivars have been introduced in a large scale to China, with a total planting area of over 100 thousand hectares (Qin et al., 2016; Su et al., 2018). The cultivated areas of the introduced olive germplasms were originally concentrated in several provinces in southern China such as Yunnan, Sichuan, Guangxi, and then gradually expended to northern China such as Gansu and Shaanxi (Su et al., 2018). Among them, Yongren region of Yunnan province is one of the primary suitable areas for olive cultivation in China and its olive industry has been strongly supported by the government in Yunnan (Su et al., 2018).

There are plenty of olive germplasm, represented by a high number of cultivars and unknown accessions (Díez et al., 2015; Mousavi et al., 2017; Belaj et al., 2018; Sion et al., 2019). The rich diversity of this species is a consequence of its allogamous nature, a remarkable tree longevity, multiple domestication events such as crosses among cultivars and local selection, as well as a lack of turnover with new breeding genotypes (Díez et al., 2015; Belaj et al., 2016; Besnard, Terral & Cornille, 2018). Since this rich germplasm represents a source of valuable traits, the identification and characterization of olive cultivars and unknown accessions is firstly required for better exploiting and protecting olive resources as well as designing breeding programs (Boucheffa et al., 2017; Cultrera et al., 2019). During the long-term cultivation of olive, wrong naming of cultivars such as homonymy or synonymy and mistakes in labeling and propagation of cultivars have often led to misleading classification and misinterpreting relationships among cultivars (Beghè et al., 2015; Mariotti et al., 2016). Furthermore, the high degree of kinship among many cultivars mainly in cases of geographic proximity and the possible appearance of clonal variation increase the difficulty of cultivar identification (Caruso, Marra & Costa, 2014; Ipek et al., 2015; Mousavi et al., 2017).

Molecular markers have been proved to be a powerful tool and employed for the identification and characterization of olive cultivars, which included microsatellites or simple sequence repeats (SSRs) (Beghè et al., 2015; Mousavi et al., 2017; Koubouris et al., 2019; Sion et al., 2019), amplified fragment length polymorphism (AFLP) (Albertini et al., 2011), restriction fragment length polymorphism (RFLP) (Bazakos et al., 2012), single nucleotide polymorphism (SNP) (Hakim et al., 2010; Belaj et al., 2012; Biton et al., 2015) and so on. Among these molecular markers, SSR markers are the most suitable and widely used for olive genotyping and cultivar discrimination due to their abundance, high polymorphism, reproducibility, and co-dominant inheritance (Baldoni et al., 2009; Beghè et al., 2015; Mousavi et al., 2017; Koubouris et al., 2019). SSR markers have also been proved to be suitable for establishing DNA fingerprinting and assessing genetic diversity, phylogenesis, population structure and phylogeography of olive cultivars (Bracci et al., 2011; Beghè et al., 2015; Hmmam et al., 2018). However, the published SSR markers are scattered and do not cover the whole olive genome. Moreover, most of the genomic-SSRs published so far are based on dinucleotide repeat microsatellites. The wide use of dinucleotide loci give rise to very close in size neighboring alleles and thus make it difficult to discriminate alleles, which may thereby cause miscalling and generate confusions (Baldoni et al., 2009; Trujillo et al., 2013; Beghè et al., 2015). Thus, the development of SSR markers with a longer core of repeat throughout the whole genome will be more informative and effective in the identification and genetic analysis of olive cultivars.

To address above-mentioned issues, this research is dedicated to: (1) develop highly informative and effective trinucleotide genomic-SSR markers, ensuring the SSR markers distributed as much as possible on the most of olive chromosomes; (2) construct the DNA fingerprints of 53 olive accessions and discriminate them, and (3) evaluate the genetic diversity and relationship of the 53 olive accessions.

## Materials and Methods

### Plant materials

A total of 53 olive accessions and *Olea europaea* subsp. *cuspidata* were analyzed in this study, which were collected from the Nuoda olive germplasm resource nursery of Yunnan Academy of Forestry and Grassland (Yongren County, Chuxiong Yi autonomous prefecture, Yunnan province, China). Among them, 50 accessions originated from six countries including Greece (10), Italy (10), Albania (5), China (16), Spain (6) and France (3), and the geographical origins of the remaining four accessions were unknown. Each accession was represented by one tree. The code, name, country of origin, and region of introduction for each accession were presented in Table 1. The olive accessions from Greece were donated by National Agricultural University of Athens, Institute of Olive Tree and Subtropical Plants of Chania, Kostelenos Olive Nurseries, and Melas-Asklipeio Olive Oil Industry in Greece, to Yunnan Academy of Forestry in 2014. The accession “Chenggu32” was selected from the seedling of “Coligno” by Forestry Bureau of Chenggu County in Shaanxi province and the “Coligno” was originated from Former Soviet Union (Li & Yu, 2012). “Chenggu32” (code 9) and “Chenggu32” (code 26) introduced from Guangyuan of Sichuan province to Nuoda olive germplasm resource nursery had same names, but they showed different phenotypic traits. The accession “Chenggu53” was selected from the seedling of “Nikitskii I” by Olive Farm of Chenggu County and the “Nikitskii I” was also originated from Former Soviet Union (Li & Yu, 2012). “Chenggu53” (code 41) and “Chenggu53” (code 22) that were respectively introduced from Wudu of Gansu province and Guangyuan to Nuoda olive germplasm resource nursery also displayed different phenotygpic traits. “Yunza No.1”, “Yunza No.2” and “Yunza No.3” are three interspecific hybrids of *Olea europaea* subsp. *europaea* var. *europaea* cv. Frantoio x *Olea europaea* subsp. *cuspidata,* belonging to a full-sib family, which were selected by Yunnan Academy of Forestry and Grassland (Ma et al., 2015; Pan et al., 2019). The accessions “Lvyuan No.1” and “Lvyuan No.8” were selected from the seedlings of mixed cultivars by Yunnan Yongren Olive Planting and Processing Company, and Yunnan Academy of Forestry and Grassland (Geng et al., 2018). The accessions “Jiufeng” and “Jiufeng No.4” were selected from the seedlings of mixed cultivars by the Hubei Research Institute of Forestry (Li & Yu, 2012; Chen et al., 2013). The accession “Ezhi No.8” was selected from the seedlings of mixed cultivars by Wuhan Botanical Garden (Li & Yu, 2012; Chen et al., 2013). The accession “Taoyuan No.1” was selected from the seedlings of mixed cultivars by Taoyuan Olive Seedling Breeding Base (Chen et al., 2013; Geng et al., 2018). The accession “Arbequina”-code 46 was represented by one tree that introduced from Greece and “Arbequina seed”-code 49 was another tree that was selected from the seedlings of “Arbequina” after natural pollination in Yunnan. Similarly, the accession “Koroneiki”-code 50 was also introduced from Greece and “Koroneiki seed”-code 43 was selected from the seedlings of “Koroneiki” after natural pollination in Yunnan.

**Table 1:** List of the 54 accessions tested in this study.

**Code of accessions**	**Name of accessions**	**Country of origin**	**Region of introduction**	**Code of accessions**	**Name of accessions**	**Country of origin**	**Region of introduction**
1	Xi No.3	Greece	Greece	33	Coratina	Italy	Wudu
2	Frantoio	Italy	Wudu	34	M2	Unknown	Yongren
3	Berat	Albania	Guangyuan	35	M4	Unknown	Yongren
4	Dritta	Italy	Dazhou	37	Ezhi No.8	China	Wudu
5	Rosciola	Italy	Dazhou	38	Kalamon	Greece	Wudu
6	Grignan	Italy	Dazhou	40	Taoyuan No.1	China	Yongsheng
8	Leccino	Italy	Wudu	41	Chenggu53	China	Wudu
9	Chenggu32	China	Guangyuan	42	Yunza No.3	China	Yunnan
10	Ottobratica	Italy	Dazhou	43	Koroneiki seed	Greece	Yunnan
11	Mixaj	Albania	Dazhou	44	Yunza No.2	China	Yunnan
12	Lucques	France	Dazhou	45	Adramittini	Greece	Greece
13	Moraiolo	Italy	Dazhou	46	Arbequina	Spain	Greece
16	Salonenque	France	Dazhou	48	Yunza No.1	China	Yunnan
17	Unnamed	China	Yongren	49	Arbequina seed	Spain	Yunnan
18	Jiufeng	China	Guangyuan	50	Koroneiki	Greece	Greece
19	Pendolino	Italy	Wudu	51	Chalkidikis	Greece	Greece
20	Kaliniot	Albania	Guangyuan	52	Jiufeng No.4	China	Guangyuan
21	Unnamed	China	Yongren	54	Lvyuan No.1	China	Yongren
22	Chenggu53	China	Guangyuan	55	Chondrolia	Greece	Greece
23	M1	Unknown	Yongren	57	Gaidourelia	Greece	Greece
25	Manzanilla de Sevilla	Spain	Yongren	58	Koutsourelia-Patrina	Greece	Greece
26	Chenggu32	China	Guangyuan	59	Koutsourelia	Greece	Greece
28	Ascolana Tenera	Italy	Wudu	61	Grossanne	Spain	Wudu
29	M3	Unknown	Yongren	98	Picual	Spain	Wudu
30	Elbasan	Albania	Guangyuan	99	Tanche	France	Wudu
31	Lvyuan No.8	China	Yongren	100	Kaliniot	Albania	Guangyuan
32	Picual	Spain	Greece	101	*Olea europaea* subsp. *cuspidata*	China	Yunnan

### DNA extraction

The DNA was isolated from silica-dried leaves after grinding using DNA secure plant kit DP320. The integrity and purity of the extracted DNA were evaluated by Thermo nano drop 2000. Before polymerase chain reaction (PCR), the DNA samples were diluted to approximately 20 ng/µl.

### SSR analysis

The complete genomic sequences of olive were retrieved from GenBank (https://www.ncbi.nlm.nih.gov/assembly/GCF_002742605.1/), with the total length of 1,141,145,264 bp. These genomic sequences were screened to search SSRs and determine their locations on the genome using the Perl script-based program, MISA (Thiel et al., 2003). The search criteria for trinucleotide, tetranucleotide, pentanucleotide, and hexanucleotide motifs were at least 5, 4, 4, and 4 repeats, respectively. The criteria for designing SSR primers were as follows: 18–24 bp in length, 40–60% GC content, 55–60 °C annealing temperature, and 100–300 bp PCR product. The SSR primers were designed by Primer Premier 5 software and then synthesized by Rui Biotech (Beijing, China). During the synthesis of primers, the universal M13 sequence (5′-TGTAAAACGACGGCCAGT-3′) was added to the 5′ end of each forward primer. Simultaneously, M13 were labeled by four fluorescent dyes (FAM, HEX, TAMRA and ROX) at the 3′ end, respectively. The labeled M13 was added to the PCR reaction to detect PCR amplification product by complementing with the unlabeled M13 added at 5′ end of primer.

PCR was performed in a volume of 20 µl containing 40–60 ng genomic DNA, 25 µmol/L of each dNTP, 2.5 unit of Taq DNA Polymerase, 10 µmol/L of forward and reverse primers, 10 µmol/L of fluorescent dyes, and 10 ×PCR buffer with 25 mmol/L Mg2+. The PCR reaction was subjected to an initial denaturation step at 94 °C for 5 min, followed by 35 cycles at 94 °C for 30 s, 56 °C for 30 s, 72 °C for 1 min, and a final elongation at 72 °C for 5 min. The PCR product was detected by capillary electrophoresis with fluorescent labeling. Considering the existence of M13 sequence (18 bp), the length of each expected fragment was obtained by subtracting 18 bp from the length of the amplified fragment.

### Data analysis

The microsatellite raw data obtained from capillary electrophoresis were analyzed by GeneMarker v2.2.0 software. The genetic diversity information parameters of each SSR locus was calculated using POPGENE 32 and Cervus v3.0.7, including the number of observed alleles (Na) and effective alleles (Ne), observed heterozygosity (Ho) and expected heterozygosity (He), Shannon’s polymorphism index (I), gene flow (Nm), null allele frequency (F(Null)), and polymorphism information content (PIC). The presence of private alleles in the 53 accessions were calculated for each SSR locus by using GenALEx v6.503 software.

The cluster analysis of 53 olive accessions was performed based on similarity coefficient using the unweighted pair group method with arithmetic (UPGMA) implemented in NTSYS-PC v2.10e. Nei’s genetic distance between the olive accessions was calculated by the PowerMarker v3.25 program, and then the principal coordinate analysis (PCoA) was conducted based on Nei’s genetic distance using GenALEx v6.503 software.

## Results

### Genome-wide identification and characterization of SSR loci

**Figure 1:**
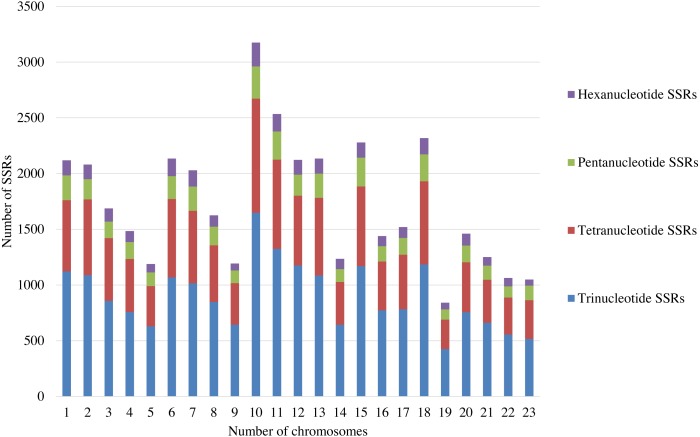
The distribution of SSRs with the different repeat types on each pair of the chromosomes.

A total of 39,953 trinucleotide, tetranucleotide, pentanucleotide, and hexanucleotide SSRs were detected by screening the whole genome of olive (2*n* = 46), with an average of around 2,000 SSRs per pair of chromosomes (Table S1). Among these SSRs, trinucleotide SSRs were the most abundant and constituted more than 51% of the total SSRs. There are about 1,000 trinucleotide SSRs in each pair of chromosomes (Fig. 1; Table S1). Thus, the trinucleotide SSRs were selected for the development of SSR markers in this study.

**Table 2:** Information on the 21 polymorphic SSR markers developed for 53 olive accessions.

**Locus**	**Primer sequence (5′–3′)**	**Repeat motif**	**Ta (°C)**	**Fluorescent labels**	**Size range (bp)**
BFU0201	F:CACTCGTCGTCAACTCCCTCG	(TTC)7	56	5′TAMRA	244–250
	R:CGATTGCTACTGCCCACTTCC				
BFU0202	F:GTTAGAACAGGAGCCACCCACC	(CCA)7	56	5′FAM	178–199
	R:GCTCCTCCAACTCATCCAAACC				
BFU0301	F:ACCGCCCAATCCTCGTCAT	(CAT)6	56	5′ROX	286–304
	R:CTTGCTGGAGAAGACAACGGA				
BFU0308	F:ACGAGGACCACTTTTTGGATTT	(ATC)5	56	5′FAM	279–357
	R:TCTGCTCCTTTACGGACGAATA				
BFU0405	F:TCCTCTCCCTAAAGTGTTTCCGA	(GAA)8	56	5′FAM	217–229
	R:TCAGGAAAAGGCTCTGCTCATCT				
BFU0510	F: AGGAAGAAGGGGATAAAGTGGG	(AGA)9	56	5′TAMRA	111–135
	R: CTTGCGGGACTTTGACGAAC				
BFU0601	F:CTCTACCTGCCAAGGCTACTGC	(ATG)6	56	5′HEX	211–268
	R:AACGGAGCAAGAACTGCCAAA				
BFU0602	F: GCCCAAACACATAACACAAACG	(AGA)6	56	5′HEX	276–315
	R: CCAAGCCGCCACCTGTTC				
BFU0707	F:TCGGTGGAAGAGTGTTCATCAA	(AAT)8	56	5′TAMRA	243–258
	R:CAGCGGTGGACCAACAGTG				
BFU0803	F: AGAGGGCATAACAGCGGTGA	(GGT)6	56	5′FAM	242–281
	R:TGTTACAATGAAGCCAAATCTGC				
BFU0808	F:CTCGGTCCCCCTATCCTCC	(TCG)5	56	5′FAM	201–216
	R:TTGCGGAATGGGAAAATGC				
BFU0902	F:TCCAAACGCAATAGGATTCAAGA	(AGA)5	56	5′FAM	283–319
	R: TATTTCTCTTTCTCGCCCCCTC				
BFU1004	F:AAGACGGACACGCTCAATAACAT	(CAT)5	56	5′HEX	238–250
	R:TGCTGGTCGCAGTCCATTATT				
BFU1008	F:TGCCTATCCGTTTCCGACAC	(AAT)5	56	5′FAM	249–261
	R:GCGTTGTCTGGTTTTCATTGG				
BFU1101	F:TGAACCAACTCATCTTCCCACC	(CCG)6	56	5′ROX	290–314
	R:ATGGGGAAATGAATGAAAGGCT				
BFU1204	F:ATCACAGCCAATAGTTCAAGCCT	(GAA)6	56	5′TAMRA	143–155
	R:TTCTCTGACTTCATACGGTGCTG				
BFU1304	F:TGTTGTGGGTTAGGTTGACTGG	(TGA)5	56	5′ROX	251–266
	R:ATTGTCAGGTTTGGGCTCATCT				
BFU1309	F:GTGATGGAGGTGGTGATTTAGAA	(GCT)5	56	5′ROX	235–247
	R:GTGCCACATTCATTCCCCA				
BFU1902	F:CAAACGGTCCCAATCCCATA	(CTC)5	56	5′TAMRA	190–217
	R:GGACTGACTGCTGGTGGCG				
BFU1908	F:GGTGAGCAACAGAACTGCGTAA	(ACT)5	56	5′FAM	205–229
	R:TCGCAAGAGGAAGTTTTGAGTC				
BFU2202	F:ACTCCAATCCAAGCGGTGC	(CCA)7	56	5′HEX	216–225
	R:CGACTGAGGTGTTCTGCTTGC				

### Development and characterization of trinucleotide SSR markers

For each pair of chromosomes, 50 trinucleotide SSR loci were selected to design primers from 1,000 trinucleotide SSR loci. A total of 1,150 SSR loci were used for primer design in the whole olive genome and only 200 SSR primer pairs were successfully designed from 23 pairs of chromosomes according to the primer design criteria above-mentioned. 143 out of the 200 SSRs were found to produce expected size of PCR products by capillary electrophoresis (Table S2), while the remaining 57 SSR markers failed to generate the expected PCR products under a series of annealing temperature. 68 out of the 143 SSRs were found to be polymorphic by further screening across eight olive accessions including “Frantoio”, “Lucques”, “Elbasan”, “Taoyuan No.1”, “Yunza No.2”, “Chalkidikis”, “Chondrolia”, and “Gorossanne”, and were then used to fingerprint the 53 olive accessions. The polymorphism information content (PIC) of the 68 SSR markers were calculated, and 24 out of 68 SSRs had PIC values higher than 0.5. 20 out of 24 SSRs were further obtained by discarding the SSRs with more than four missing data and most of 20 SSRs had none or only one missing data. It was noted that one SSR marker (BFU1309) had a PIC value of 0.44, but it specifically distinguished two accessions (“Grignan” and “Leccino”). Therefore, 21 SSR markers including BFU1309 were used for the further genetic analysis. These SSR markers covered most chromosomes of olive (Table 2), which well discriminated the 53 olive accessions.

The observed number of alleles (Na) varied from three (BFU0201, BFU1004, BFU2202) to ten (BFU0602), and a total of 108 alleles were detected in the analyzed accessions with an average of 5.14 alleles per locus. The average of observed heterozygosity (Ho) and expected heterozygosity (He) was 0.52 and 0.67, respectively. The polymorphism information content (PIC) ranged from 0.44 (BFU1309) to 0.79 (BFU0803) with an average of 0.61. There were up to 20 pair of SSR markers with PIC value higher than 0.5, indicating that these markers had a high level of polymorphism. Other genetic diversity parameters, such as the Shannon’s information index (I) and gene flow (Nm), were well correlated with the PIC, Na, and Ho (Table 3).

It was noted that a total of 31 private alleles were detected in 15 olive accessions, and the number of private alleles varied from one to six among accessions. The “Yunza No.1”, “Yunza No.2”, and “Yunza No.3” had five or six private alleles, and the remaining accessions had almost one private alleles (Table 4). Among 31 private alleles, five unique alleles were detected in four accessions including “Gaidourelia”, “Ascolana Tenera”, “Kaliniot”, and “Salonenque”.

### Establishment of DNA fingerprints

The DNA fingerprints of 53 olive accessions was established based on the bands amplified by 21 polymorphic primer pairs. The size of amplified bands was determined by the DNA molecular weight standard, which was used for representing allelic variation of each SSR locus. According to the chromosome order in the olive genome, 21 SSR loci were serially arranged to form the DNA fingerprints of 53 olive accessions (Supplemental Information 1, Table S3). A minimum of four pairs of primers (BFU0803, BFU0510, BFU1908, BFU1309) could discriminate all 53 accessions.

### UPGMA cluster analysis based on similarity coefficient

The similarity matrice for the 53 olive accessions and *O. europaea* subsp. *cuspidata* was built using NTSYS-PC v2.10e. The genetic similarity coefficient among the 53 accessions ranged from a maximum of 0.99 between “Mixaj” and “Ottobratica” to a minimum of 0.48 between “YunzaNo.2” and “Chalkidikis”, with an overall average of 0.69 (Table S4).

**Figure 2:**
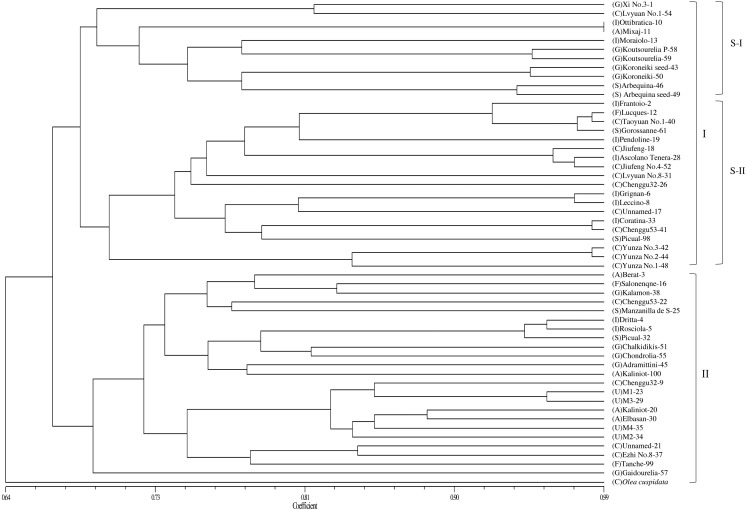
A dendrogram of genetic relationship among 53 olive accessions and *O. europaea* subsp. *cuspidata* based on 21 SSR markers. Capital letters in parentheses are the initials of countries that represent the origin of olive accessions. A, Albania; C, China; F, France; G, Greece; I, Italy; S, Spain; U, Unknown.

Based on the genetic similarity coefficients, a dendrogram for the 53 accessions and *O. europaea* subsp. *cuspidata* was constructed using the UPGMA clustering analysis (Fig. 2). The male parent of “Yunza No.1”, “Yunza No.2” and “Yunza No.3” (*O. europaea* subsp. *cuspidata*), as a separate branch, was not closely related to the 53 olive accessions (Fig. 2). The 53 olive accessions were classified into two main groups at the similarity coefficient of 0.66. 30 accessions were clustered in Group I and 23 accessions were clustered in group II. Group I was further divided into two subgroups including subgroup I and II. Subgroup I contained 11 accessions. Subgroup II included 19 accessions, and “Yunza No.1”, “Yunza No.2”, “Yunza No.3” as well as their female parent (“Frantoio”) were clustered in this subgroup. It is worth remarking that some accessions clustered in a same clade were from same geographical origin, such as “Jiufeng” and “JiufengNo.4” from China, “Grignan” and “Leccino” from Italy, while the other accessions in a same clade were from different geographical origin, such as “Coratina” and “Chenggu53–41” from Italy and China, “Lucques”, “Grossanne” and “TaoyuanNo.1” from France, Spain and China, respectively. For group II, “Gaidourelia” is different from the remaining 22 accessions.

**Table 3:** Genetic diversity parameters for 21 SSR loci of 53 olive accessions.

**Locus**	**Na**	**Ne**	**Ho**	**He**	**I**	**Nm**	**F(Null)**	**PIC**	**Discriminating Power(**the numbers of different allele combination types**)**
BFU0201	3	2.84	0.48	0.65	1.07	2.54	0.14	0.57	6
BFU0202^a^	6	3.49	0.85	0.72	1.46	2.47	0.10	0.68	12
BFU0301	5	2.70	0.14	0.62	1.20	1.45	0.58	0.57	8
BFU0308	6	2.49	0.36	0.57	1.17	1.40	0.04	0.52	9
BFU0405	5	2.76	0.68	0.64	1.16	1.43	0.02	0.58	9
BFU0510^a^	6	4.62	0.85	0.79	1.61	1.79	0.03	0.75	14
BFU0601	4	3.09	0.85	0.68	1.18	3.30	0.11	0.61	6
BFU0602^a^	10	4.36	0.46	0.77	1.74	1.67	0.24	0.73	14
BFU0707	4	2.91	0.41	0.67	1.17	1.79	0.24	0.60	9
BFU0803^a^	7	5.23	0.91	0.81	1.83	1.96	0.03	0.79	16
BFU0808	4	2.93	0.26	0.66	1.12	2.40	0.35	0.59	6
BFU0902^a^	6	3.55	0.42	0.73	1.43	0.70	0.27	0.68	13
BFU1004	3	2.57	0.26	0.62	1.02	1.55	0.41	0.54	6
BFU1008	4	2.69	0.20	0.62	1.17	0.51	0.51	0.57	7
BFU1101^a^	6	3.59	0.42	0.73	1.43	1.58	0.27	0.68	12
BFU1204	4	2.47	0.44	0.60	1.04	2.25	0.14	0.52	7
BFU1304	4	3.35	0.69	0.71	1.30	1.65	0.04	0.66	8
BFU1309	5	2.05	0.47	0.52	1.05	1.92	0.01	0.44	9
BFU1902^a^	7	3.59	0.77	0.72	1.47	2.25	0.04	0.67	12
BFU1908	6	2.74	0.79	0.64	1.25	3.19	0.12	0.58	7
BFU2202	3	2.66	0.25	0.64	1.03	0.60	0.43	0.56	5
Total	108								
Mean	5.14	3.18	0.52	0.67	1.28	1.83	0.20	0.61	9.29

**Notes.**

Na observed number of alleles Ne effective number of alleles Ho observed heterozygosity He expected heterozygosity I Shannon’s Information index Nm gene flow F (Null) null allele frequency PIC polymorphism information content

a The most informative SSR markers.

**Table 4:** List of olive accessions with one or more private alleles.

**Code of accessions**	**Name of accessions**	**Country of origin**	**No. Loci with private alleles**	**Loci with private alleles**					
1	XiNo.3	Greece	1	BFU0405					
10	Ottobratica	Italy	1	BFU0602					
16	Salonenque	France	1	BFU0902					
20	Kaliniot	Albania	1	BFU0808					
28	Ascolana Tenera	Italy	1	BFU1908					
33	Coratina	Italy	1	BFU0707					
34	M2	Unknown	1	BFU1101					
35	M4	Unknown	1	BFU0602					
41	Chenggu53	China	1	BFU0707					
42	YunzaNo.3	China	6	BFU0405	BFU0510	BFU0601	BFU0707	BFU1101	BFU1902
44	YunzaNo.2	China	6	BFU0405	BFU0510	BFU0601	BFU0707	BFU1101	BFU1902
48	YunzaNo.1	China	5	BFU0405	BFU0601	BFU0707	BFU1101	BFU1902	
49	Arbequina seed	Spain	1	BFU0308					
57	Gaidourelia	Greece	3	BFU0301	BFU1204	BFU1902			
58	Koutsourelia-Patrina	Greece	1	BFU0308					
**Total**	15		31						

### Principal coordinate analysis

**Figure 3:**
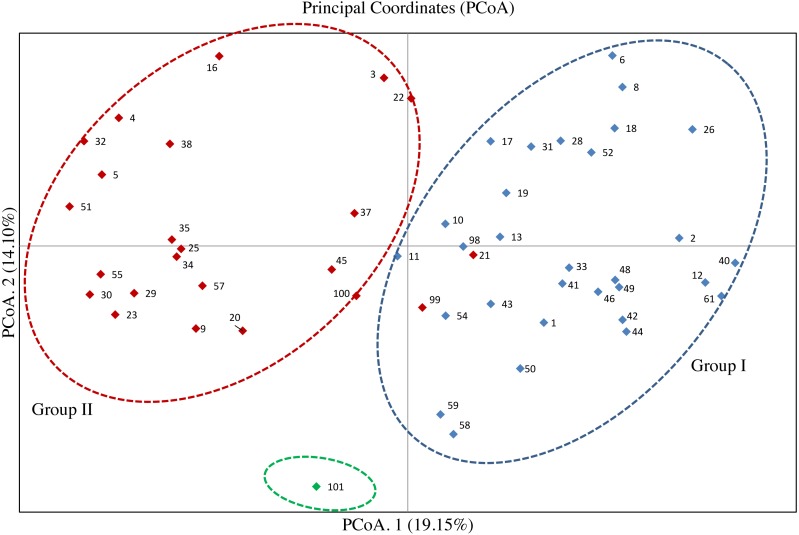
Principal coordinate analysis (PCoA) of 53 olive accessions and *O. europaea* subsp. *cuspidata* based on 21 SSR markers.

The PCoA for the 53 olive accessions and *O. europaea* subsp. *cuspidata* based on Nei’s genetic distance was shown in Fig. 3. The results showed that the first two principal coordinates explained about 33.25% of the total genetic variation among tested accessions, of which 19.15% attributed to the first coordinate and 14.10% to the second one, respectively. Except for the *O. europaea* subsp. *cuspidata,* the 53 olive accessions were classified into two groups. 32 olive accessions were gathered in Group I, and 21 accessions were gathered in Group II. The results of PCoA for the 53 olive accessions was basically in agreement with that of the UPGMA cluster analysis.

## Discussion

During the cultivation and collection of the olive, the occurrence of confusion between cultivars and the emergence of clonal variation make it difficult to discriminate or distinguish the cultivars (Beghè et al., 2015; Dridi et al., 2018; Koubouris et al., 2019; Sion et al., 2019). Some molecular markers have been employed in the identification of olive cultivars in recent years. Several studies revealed that SSR markers are more suitable for the identification and genetic variation analysis of olive cultivars than SNP markers because the former has higher mutation rate than the latter (Biton et al., 2015; Belaj et al., 2018).The SSR markers of olive were originally developed by Sefc et al. (2000), Carriero et al. (2002) and Cipriani et al. (2002), and they distinguished 12–47 olive accessions using 15–30 pairs of polymorphic SSR primers based on dinucleotide repeats. These dinucleotide genomic-SSR markers were then extensively used for the identification of more olive accessions in later researches (Beghè et al., 2015; Lazović et al., 2016; Dridi et al., 2018; Boucheffa et al., 2019). However, dinucleotide SSR markers produce less easily detected differences in the length of alleles, thereby increasing allele miscalling compared with a longer core of repeats such as trinucleotide SSR markers (Diwan & Cregan, 1997; Song, Fickus & Cregan, 2002). Trinucleotide SSR markers have been demonstrated to be highly polymorphic and stably inherited in soybean and wheat (Cregan et al., 1999; Song, Fickus & Cregan, 2002). Genomic-SSR markers based on trinucleotide repeats have not been developed so far in olive. In this study, a new set of highly polymorphic trinucleotide SSR markers were successfully developed, covering most chromosomes of olive (Table 2). This work provides a powerful tool for a proper management of olive accessions introduced in China in recent years, thereby avoiding management problems detected in traditional olive growing countries. Additionally, the genetic diversity of olive accessions could be assessed based on newly developed SSR markers, and thus it is possible to select certain cultivars for hybridization to achieve new olive cultivars with potential usefulness.

In recent years, DNA fingerprints have been successively established in many plants, such as chrysanthemum, oil camellia, durian, and pistachio (Zhang et al., 2014; Chen et al., 2016; Siew et al., 2018; Mannino, Gentile & Maffei, 2019). In the olive, DNA fingerprinting is considered very important because both the productivity and quality of olive products are intrinsic characteristics of the original cultivars (Muzzalupo et al., 2018). For example, in Croatia Istria, the DNA fingerprints of 27 olive accessions clarified the genetic relationships between native and introduced varieties (Poljuha et al., 2008). For the olive germplasm in Montenegro, the DNA fingerprints provided evidence that olive plants were propagated by cuttings or seedlings rather than by grafting (Lazović et al., 2016).

The values of genetic diversity parameters indicated a high polymorphism of the 21 trinucleotide genomic-SSR markers. The average number of alleles per locus is similar to, or higher than that reported by Carriero et al. (2002) and Cipriani et al. (2002), which can be affected by many factors, such as the number of accessions, the geographical origin of cultivars, and the different loci investigated (Lopes et al., 2004). PIC represents the degree of microsatellite variation and evaluates the discriminatory power of SSR markers (Nachimuthu et al., 2015), which is not affected by the above-mentioned factors (Delgado-Martinez et al., 2012). In this study, the average value of PIC was 0.51 (Table 3), indicating a high degree of polymorphism among the 21 SSR markers according to the criteria described by Botstein et al. (1980). The observed heterozygosity (Ho) and expected heterozygosity (He) indices can reveal the genetic variability within the species (Delgado-Martinez et al., 2012). The average Ho for the 21 SSR markers was 0.52 and represented a high degree of genetic variability among the 53 accessions. In several previous reports, the average Ho was higher than 0.5 based on around ten SSR markers in the olive (Lazović et al., 2016; Mousavi et al., 2017; Dridi et al., 2018; Boucheffa et al., 2019). This phenomenon indicates that the average value of Ho is influenced by the number of SSR markers to some extent. The observed heterozygosity (Ho) of some loci was lower than the expected heterozygosity (He) (Table 3), which are considered to be interfered by an excess of homozygotes or implied the presence of null allele (Cipriani et al., 2008; Hmmam et al., 2018). For example, some loci (BFU0301, BFU1008, BFU2202) with a high value of null allele frequency (F (Null)) indicated an excess of homozygotes rather than presenting a large number of null alleles (Table S3). For a null allele, its presence was due to a mutation (insertion/deletion) on the primer binding site that thus caused variation in the flanking sequence of SSR locus (Jones & Ardren, 2003; Noormohammadi et al., 2014). Based on the values of PIC, the discriminating power and other genetic diversity parameters, seven SSR markers including BFU0803, BFU0510, BFU0602, BFU0202, BFU1902, BFU0902 and BFU1101 were classified as the most informative SSR markers (Table 3), which could distinguish most of the 53 accessions. The remaining 14 SSR markers, as the minor informative SSR markers, were also indispensable for the identification of some certain accessions in this study. Among the 14 SSR markers, only one SSR marker (BFU1309) distinguished “Grignan” and “Leccino” (Table S3; raw data file). However, it could not be ruled out that there are other SSRs in the olive genome that could distinguish the two olive accessions, because only 1150 trinucleotide SSR loci were selected from the olive genome to design SSR primers in this study.

The presence of private alleles could reflect the genetic diversity of the germplasm to some extent and facilitates the identification of accessions (Mariotti et al., 2016; Boucheffa et al., 2017), which would be valuable in future breeding endeavors (Boucheffa et al., 2017). In this study, private alleles were found in 15 olive accessions, of which “Yunza No.1”, “Yunza No.2”, “Yunza No.3” from China contained more private alleles than the other 12 accessions (Table 4). Considering that the three “Yunza” accessions are interspecific hybrids of *O. europaea* subsp. *europaea* var. *europaea* cv. Frantoio x *O. europaea* subsp. *cuspidata*, it could be explained that more private alleles were distributed in these three accessions and displayed a wider genetic variability. However, three private alleles were detected in “Gaidourelia”, which might be caused by more gene exchange or possible mutation during its domestication from Greece to Yunnan, China.

The analysis of genetic variation based on SSR markers can clearly uncover the genetic relationship of olive accessions (Beghè et al., 2015). In this study, the dendrogram clearly separated all the olive accessions into two different groups, which was supported by the results of PCoA (Figs. 2 and 3), thereby confirming the effectiveness of the 21 SSR markers. The separation of olive accessions is consistent with their geographical origins and genetic background to some extent (Boucheffa et al., 2019). The olive is originally present in the eastern Mediterranean coast and then gradually expands to the central and west Mediterranean basin (Vossen, 2007; Díez et al., 2015). The migratory history of olive is particularly complicated, especially in the central Mediterranean basin where the occurrence of second and separate domestication of olive resulted in frequent genetic exchanges between olives (Díez et al., 2015), which can reasonably explain why the genetic relationship of some olive accessions did not well correspond to geographical origins in this study. For example, “Koroneiki” and “Arbequina” were from Greece and Spain, but clustered in the same clade and displayed relatively close genetic distances (Figs. 2 and 3). However, both “Arbequina” and “Picual” were from Spain, but clustered in two different subgroups and displayed relatively distant genetic distances (Figs. 2 and 3). Several studies have also found similar phenomena (Noormohammadi et al., 2014; Biton et al., 2015; Mousavi et al., 2017), which further support our results. This phenomenon of genetic variation within a population may probably attributed to the gene flow among different olive cultivars. The gene flow can be caused by domestication, introduction, hybridization and other related breeding manipulations between olive cultivars (Breton, Tersac & Bervillé, 2006). Moreover, globalization has intensified the movement of olive cultivars between countries, thereby strengthening the gene flow (Trujillo et al., 2013). Several traditional olive cultivars such as “Frantoio”, “Pendoline”, “Coratina”, “Ottobratica” and “Ascolana Tenera” clustered into a same subgroup, exhibiting a close genetic relationship consistent with previous studies (Biton et al., 2015; Mousavi et al., 2017; Di Rienzo et al., 2018; Cultrera et al., 2019). Additionally, the genetic relationship of 11 accessions was previously reported based on the published SSRs, including “Frantoio”, “Coratina”, “Leccino”, “Pendoline”, “Ascolana Tenera”, “Koroneiki”, “Kalamon”, “Grossanne”, “EzhiNo.8”, “Chenggu32”, and “Chenggu53” (Qin et al., 2016). For these 11 accessions, the result of this study was basically consistent with the previous result except for two accessions (“Coratina” and “EzhiNo.8”). The clustering analysis showed the occurrence of several homonymy cases in the tested 53 olive accessions, as a consequence of confusion between accessions. For example, two accessions with a same name of “Koroneiki ” corresponding to code 50 and code 43 gathered in the same subgroup with a high genetic similarity coefficient, and so did the “Arbequina (codes 46 and 49)” (Table 1, Table S4, Figs. 2 and 3). The results were consistent with the previous report about the genetic analysis of “Koroneiki” and “Arbequina” based on ISSR markers (Chen et al., 2013). Given that the “Arbequina seed”-code 49 and “Koroneiki seed”-code 43 were respectively selected from the seedlings of “Arbequina”-code 46 and “Koroneiki”-code 50 after natural pollination, it was not unexpected that slight genetic differences were found between the two accessions of “Arbequina” or “Koroneiki”. However, the other four homonymous accessions including “Chenggu53 (codes 22 and 41)”, “Chenggu32 (codes 9 and 26)”, “Kaliniot (codes 20 and 100)”, and “Picual (codes 32 and 98)” displayed high degree of genetic differentiation within two accessions with the same name (Figs. 2 and 3). Similar findings were revealed for the four homonymous accessions based on previous published SSR markers (Li & Yu, 2012; Geng et al., 2018). The genetic similarity coefficient between each pair of homonymous accessions was close to or lower than the average value of the similarity coefficient (Table S4), which suggested the inter-cultivar variability between these homonymous accessions. This homonymous phenomenon is probably caused by the error during the propagation of olive cultivars or the mislabeling in olive nurseries and germplasm banks (Koubouris et al., 2019). For the four accessions of unknown geographical origin, “M1”, “M2”, “M3”, and “M4” were clustered in a subgroup of only seven accessions, together with “Chenggu 32” (code 9) form China, “Kaliniot” (code 20) and “Elbasan” from Albania (Fig. 2). As above-mentioned, two homonymy cases occurred in “Chenggu 32” (codes 9 and 26) and “Kaliniot” (codes 20 and 100), and the two accessions of “Chenggu 32” or “Kaliniot” were clustered in two different subgroups, which made it difficult to accurately determine their names. Thus, it is currently determined that “M1”, “M2”, “M3”, and “M4” with unknown origin were closely related to “Elbasan” from Albania in the genetic relationship.

## Conclusions

In conclusion, a new set of highly polymorphic trinucleotide genomic-SSR markers for olive was successfully developed in this study. The developed 21 SSR markers well discriminated 53 olive accessions. DNA fingerprints were constructed for 53 accessions based on 21 SSR markers. The genetic characterization and relationships of the 53 olive accessions were revealed. The results demonstrated that the newly developed 21 SSR markers are reliable and useful for the identification of more olive accessions and genetic analysis, which provided important information for the breeding program and germplasm preservation of olive. The acquisition of reference materials from well-known international Olive Germplasm Collections will provide an improvement for future works.

##  Supplemental Information

10.7717/peerj.8573/supp-1 Table S1 The distribution of SSRs with the different repeat type in the whole genome of olive

10.7717/peerj.8573/supp-2 Table S2 Information of the 143 SSR markers

10.7717/peerj.8573/supp-3 Table S3 The DNA fingerprints of 53 olive accessions based on 21 SSR markers

10.7717/peerj.8573/supp-4 Table S4 The genetic similarity coefficient among 53 olive accessions based on 21 SSR markers

10.7717/peerj.8573/supp-5 Supplemental Information 1 Amplified fragment length based on 21 SSR loci of 53 olive accessions

10.7717/peerj.8573/supp-6 Supplemental Information 2 The outputs of 21 SSRs on automatic sequencer for 53 accessions

## Additional Information and Declarations

### Data Availability

The following information was supplied regarding data availability: The raw measurements are available in the Supplementary Filess.
