# Supplementary material for: The newly developed genomic-SSR markers uncover the genetic characteristics and relationships of olive accessions
Source: PeerJ. 2020 Feb 13;8:e8573. doi: 10.7717/peerj.8573 (PMC7024576; doi:10.7717/peerj.8573)
Supplement: Table S1 [file peerj-08-8573-s001.docx]

| Number of chromosomes | Trinucleotide  SSRs | Tetranucleotide  SSRs | Pentanucleotid  SSRs | Hexanucleotide  SSRs | Total |
| --- | --- | --- | --- | --- | --- |
| 1 | 1122 | 639 | 223 | 135 | 2119 |
| 2 | 1088 | 680 | 182 | 131 | 2081 |
| 3 | 857 | 563 | 149 | 118 | 1687 |
| 4 | 757 | 477 | 152 | 98 | 1484 |
| 5 | 629 | 361 | 123 | 75 | 1188 |
| 6 | 1068 | 704 | 205 | 157 | 2134 |
| 7 | 1015 | 651 | 218 | 145 | 2019 |
| 8 | 848 | 508 | 168 | 101 | 1625 |
| 9 | 644 | 372 | 114 | 63 | 1193 |
| 10 | 1648 | 1024 | 289 | 215 | 3176 |
| 11 | 1324 | 801 | 253 | 156 | 2534 |
| 12 | 1175 | 626 | 189 | 133 | 2123 |
| 13 | 1085 | 697 | 218 | 135 | 2135 |
| 14 | 643 | 384 | 116 | 92 | 1235 |
| 15 | 1169 | 716 | 258 | 136 | 2279 |
| 16 | 773 | 438 | 138 | 90 | 1439 |
| 17 | 782 | 490 | 151 | 97 | 1520 |
| 18 | 1185 | 746 | 241 | 146 | 2318 |
| 19 | 425 | 264 | 92 | 60 | 841 |
| 20 | 756 | 448 | 150 | 106 | 1460 |
| 21 | 661 | 386 | 127 | 77 | 1251 |
| 22 | 555 | 333 | 100 | 75 | 1063 |
| 23 | 516 | 348 | 130 | 55 | 1049 |
| Total | 20725 | 12656 | 3986 | 2596 | 39953 |
| Mean | 901 | 550 | 332 | 113 | 1737 |

Table S1. The distribution of SSRs with the different repeat type in the whole genome of olive
