# Supplementary figures and images for: The newly developed genomic-SSR markers uncover the genetic characteristics and relationships of olive accessions
Source: PeerJ. 2020 Feb 13;8:e8573. doi: 10.7717/peerj.8573 (PMC7024576; doi:10.7717/peerj.8573)

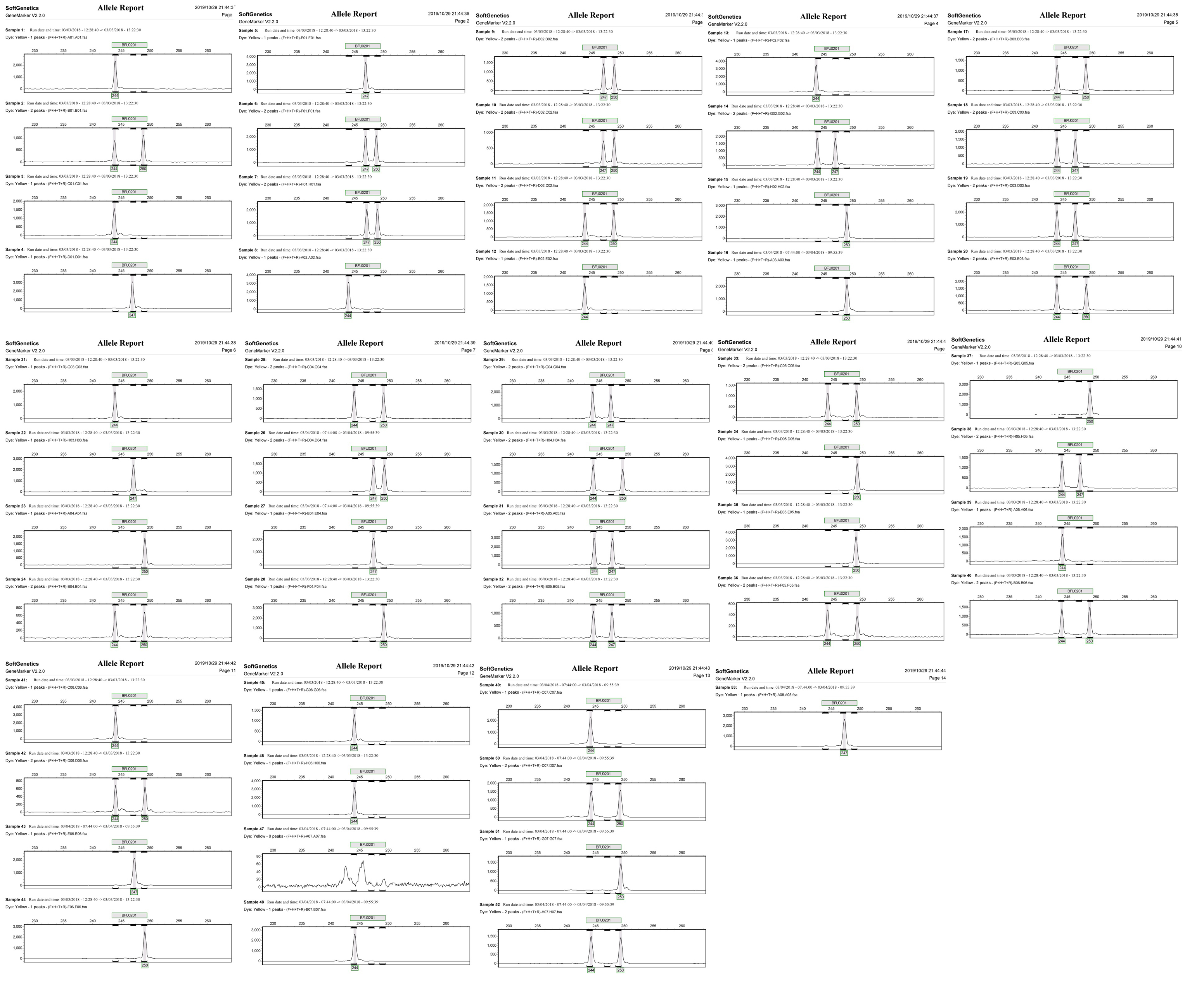

Supplement: Supplemental Information 2 [file peerj-08-8573-s006.zip › Peak maps/BFU0201.jpg]

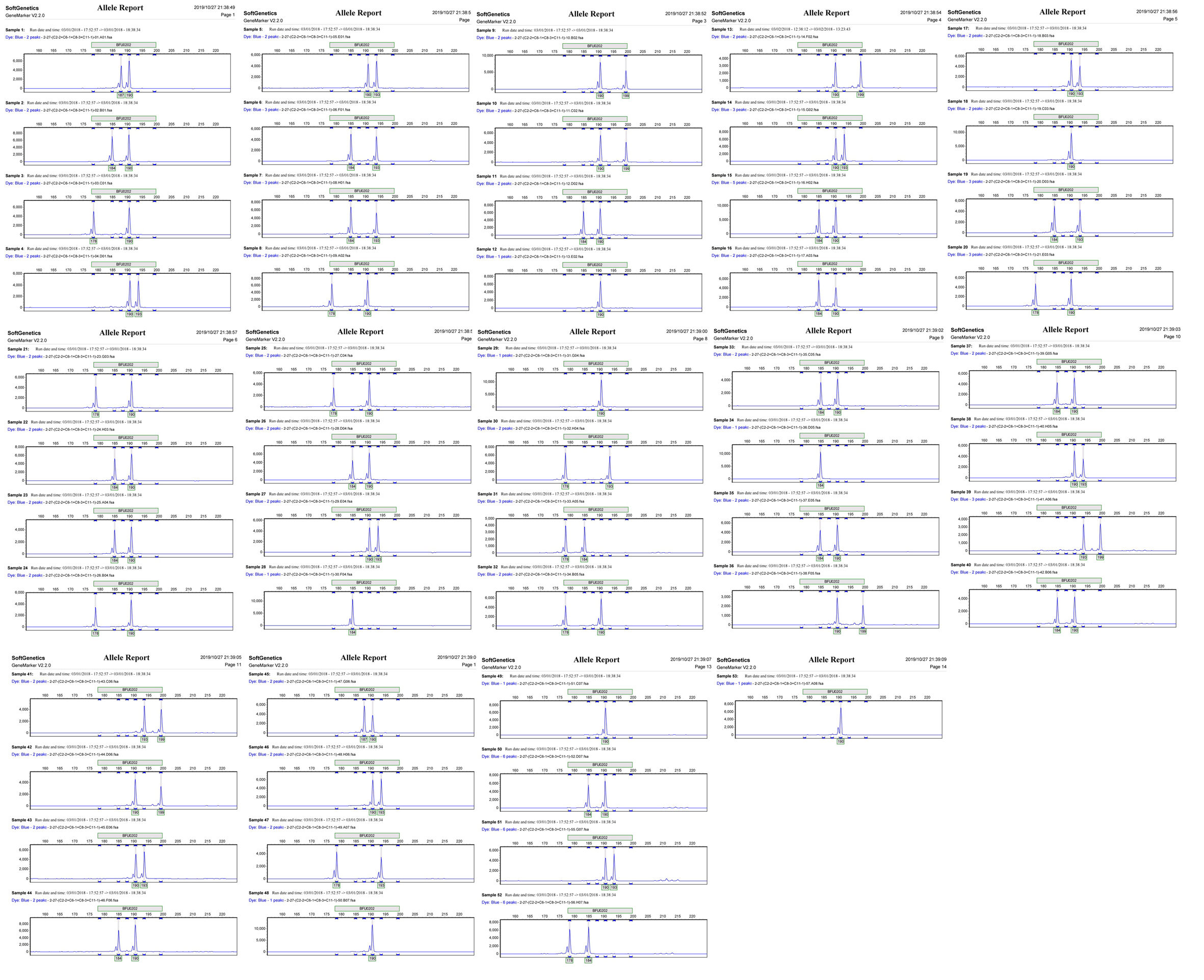

Supplement: Supplemental Information 2 [file peerj-08-8573-s006.zip › Peak maps/BFU0202.jpg]

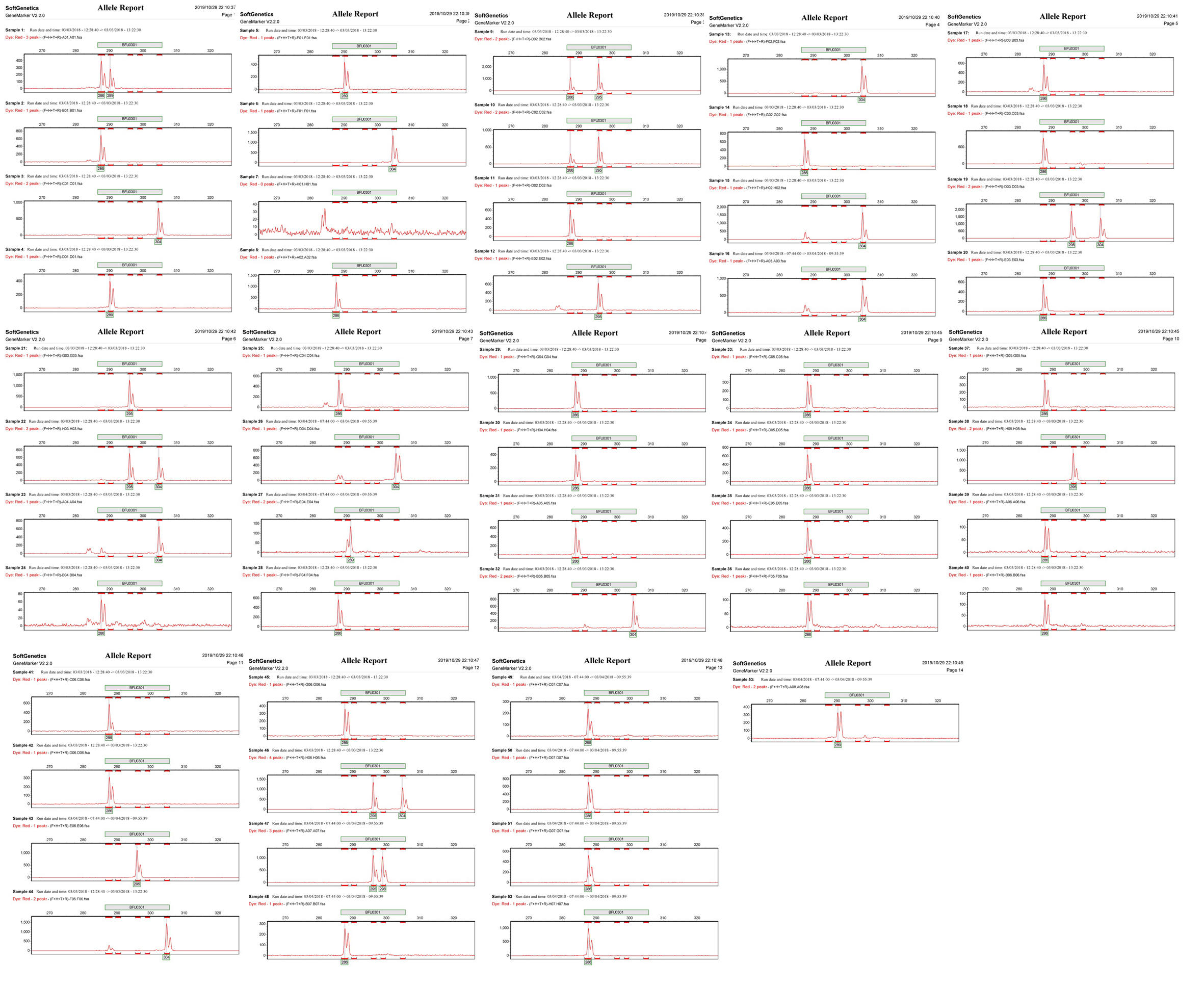

Supplement: Supplemental Information 2 [file peerj-08-8573-s006.zip › Peak maps/BFU0301.jpg]

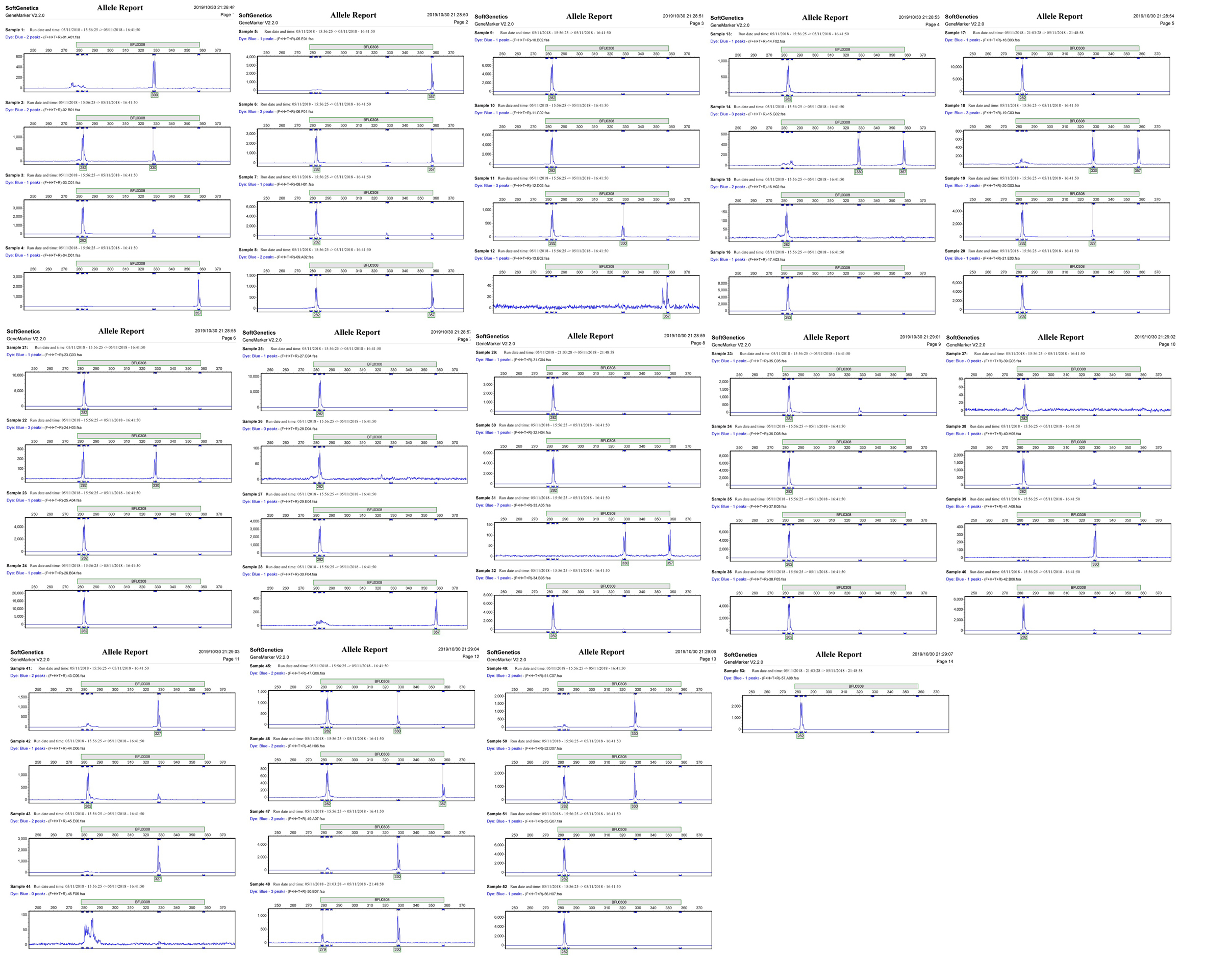

Supplement: Supplemental Information 2 [file peerj-08-8573-s006.zip › Peak maps/BFU0308.jpg]

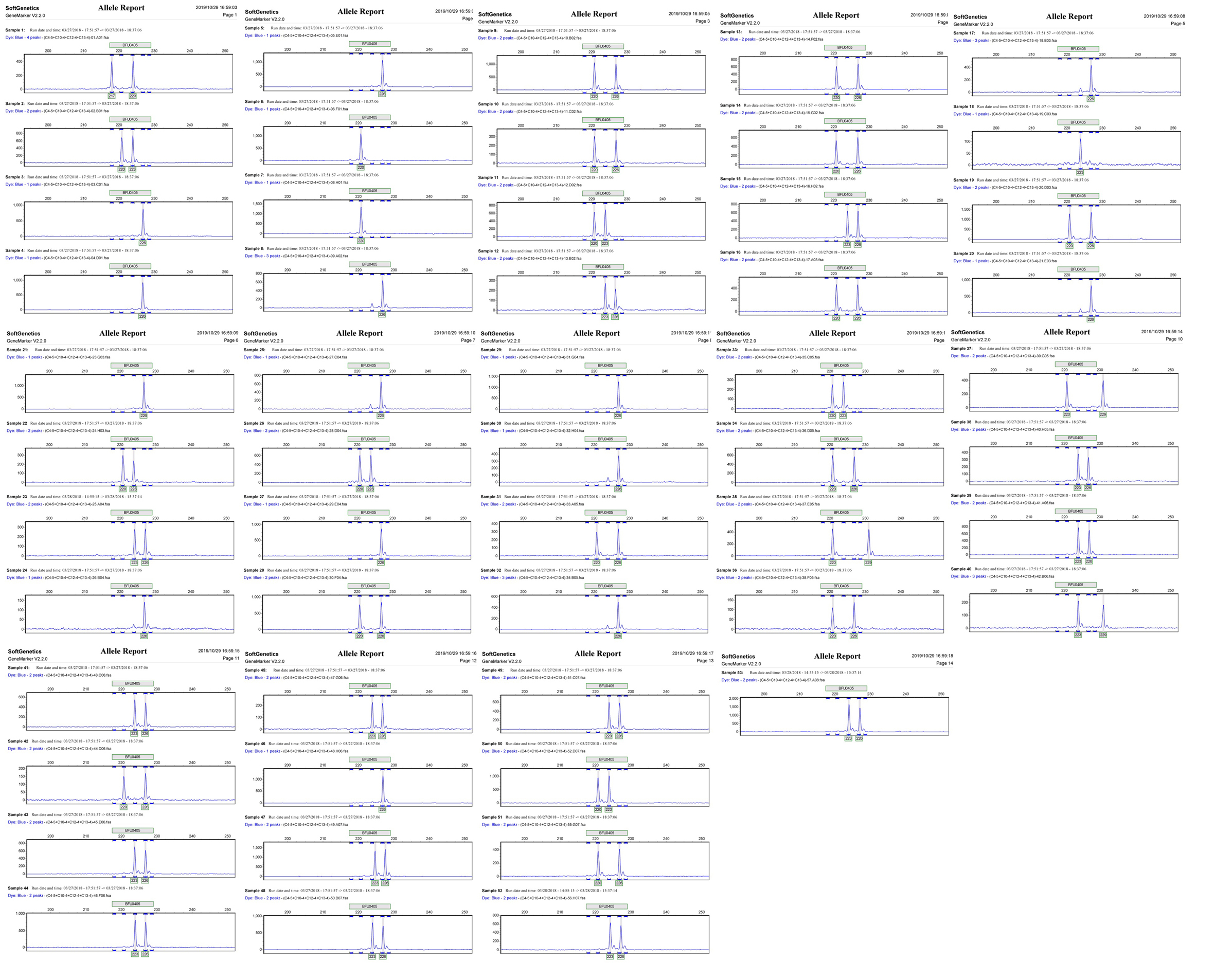

Supplement: Supplemental Information 2 [file peerj-08-8573-s006.zip › Peak maps/BFU0405.jpg]

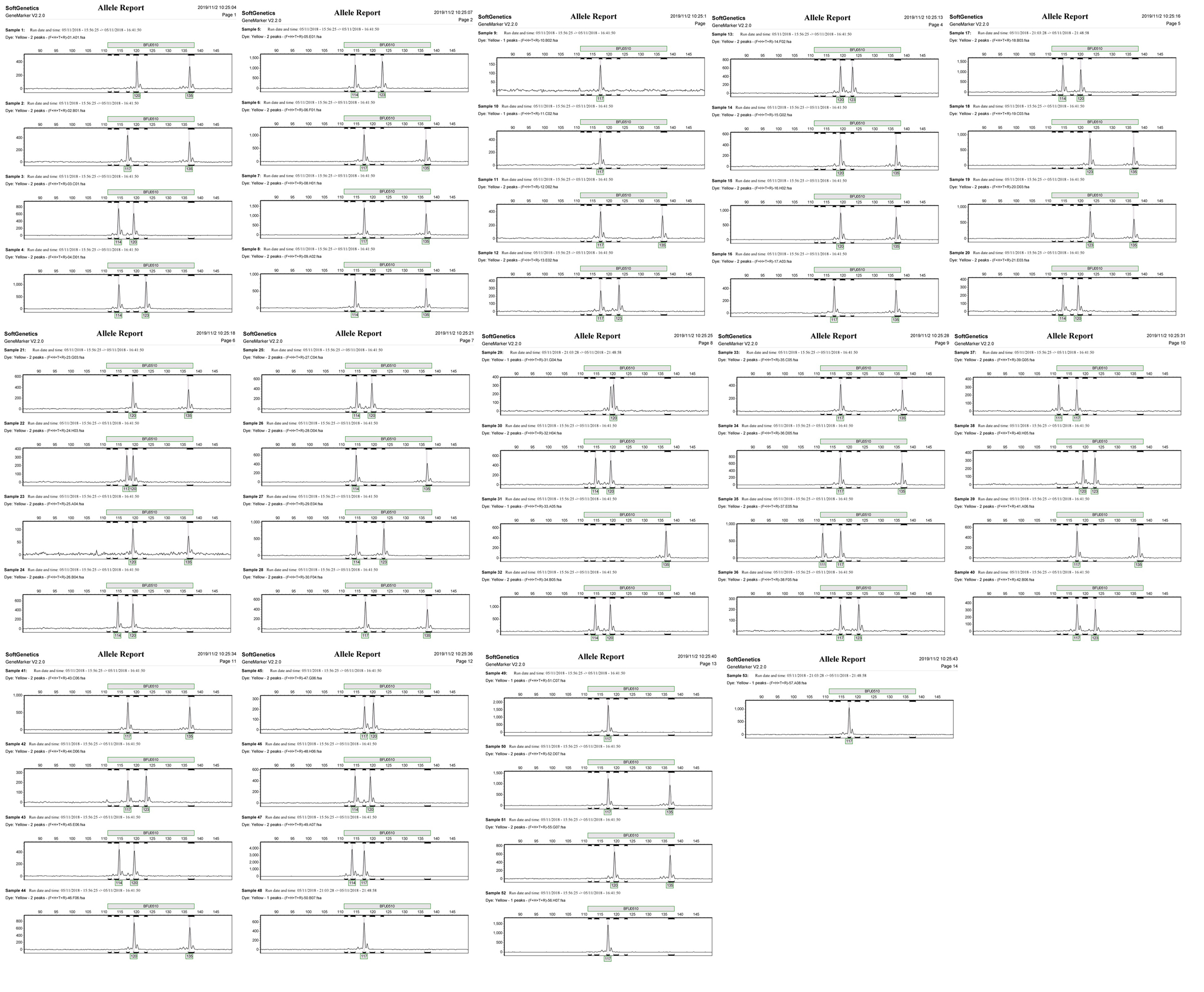

Supplement: Supplemental Information 2 [file peerj-08-8573-s006.zip › Peak maps/BFU0510.jpg]

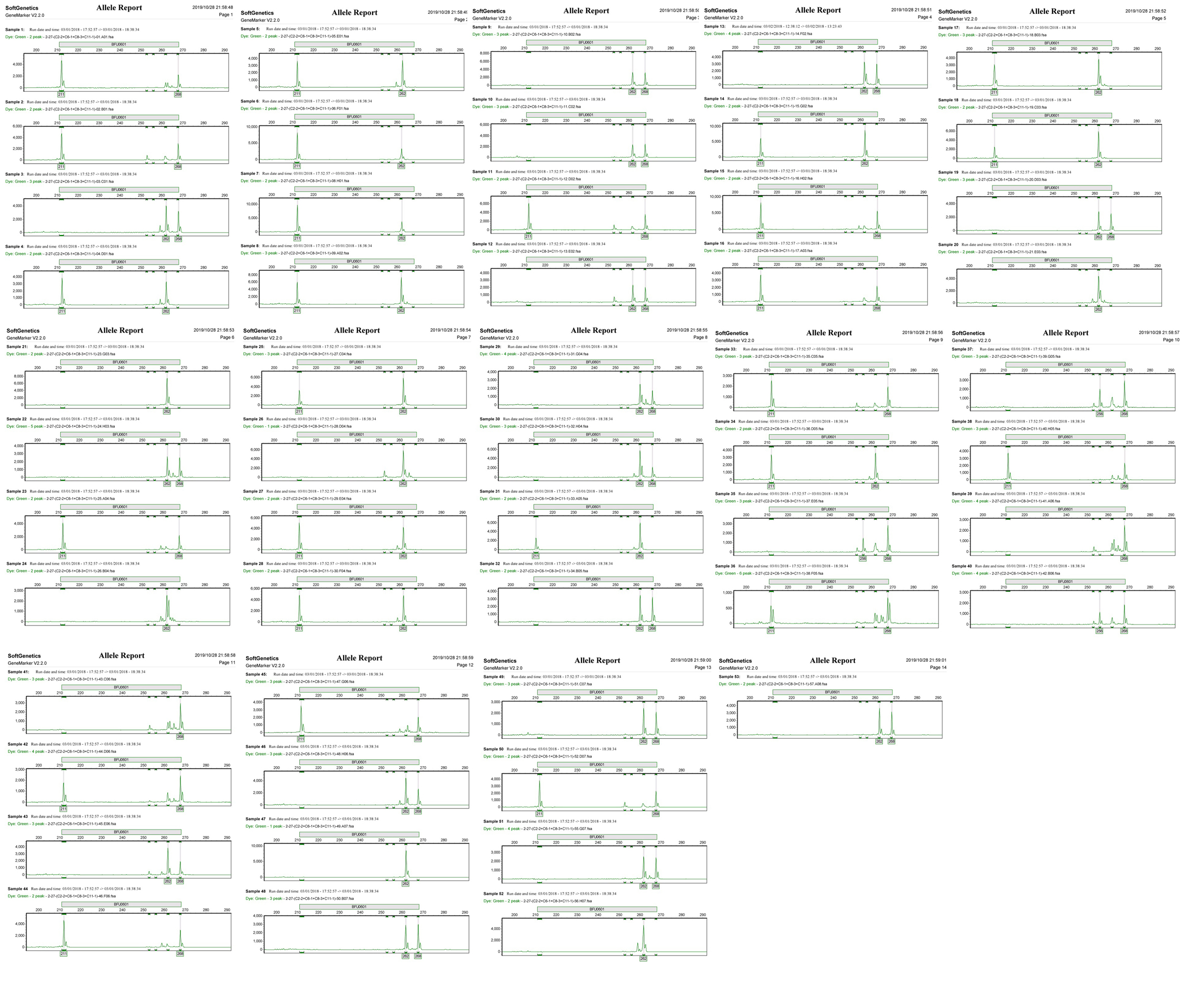

Supplement: Supplemental Information 2 [file peerj-08-8573-s006.zip › Peak maps/BFU0601.jpg]

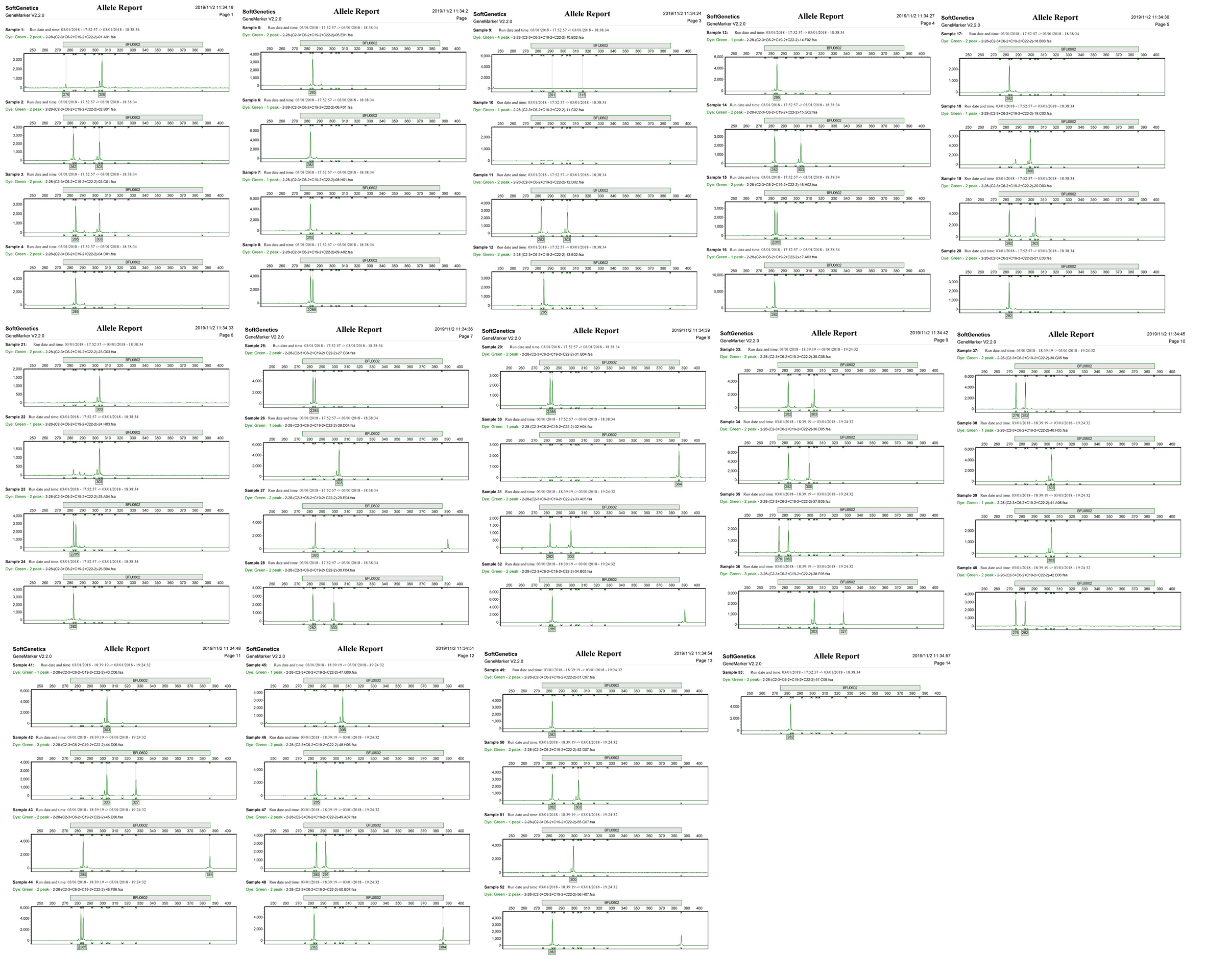

Supplement: Supplemental Information 2 [file peerj-08-8573-s006.zip › Peak maps/BFU0602.jpg]

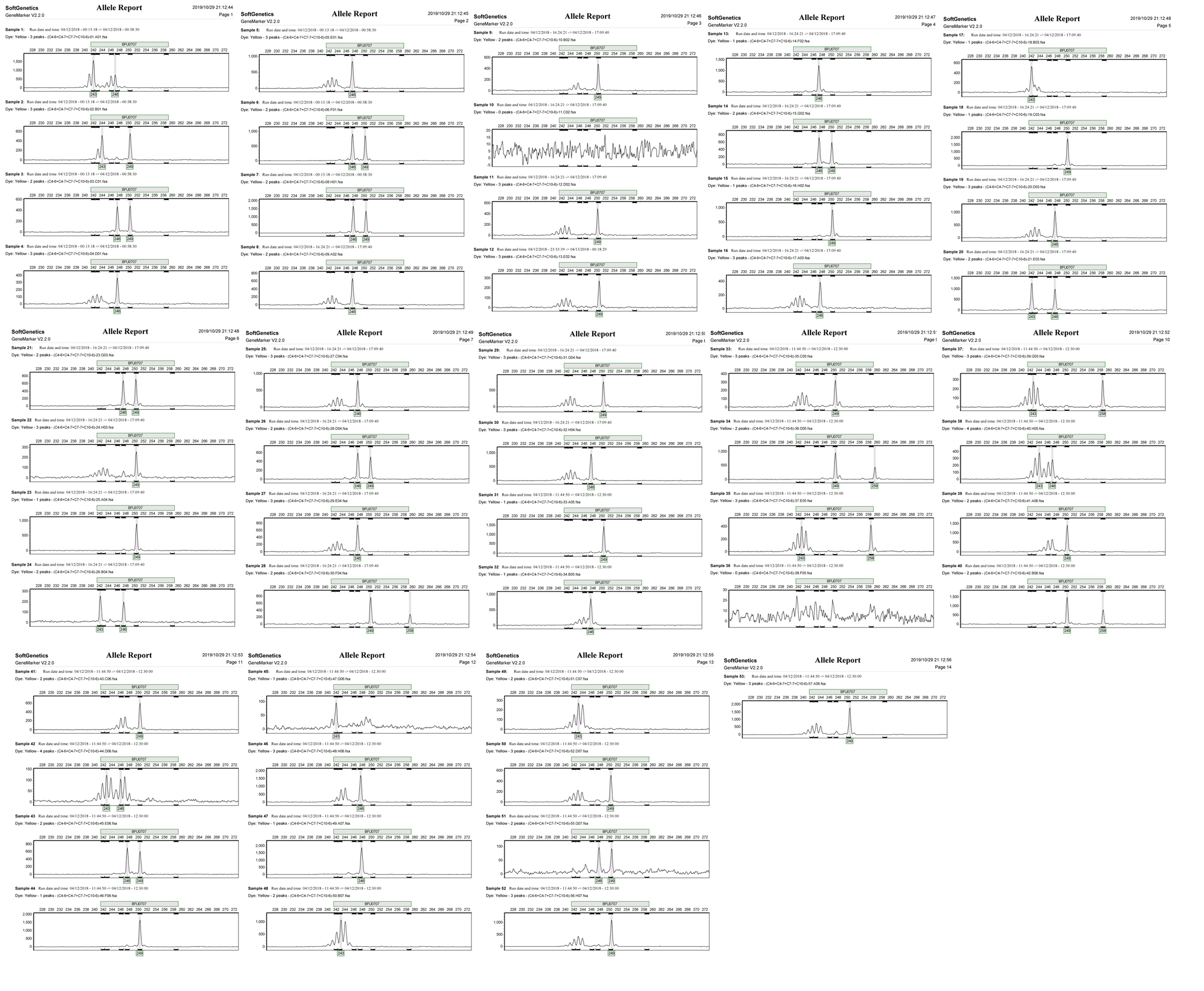

Supplement: Supplemental Information 2 [file peerj-08-8573-s006.zip › Peak maps/BFU0707.jpg]

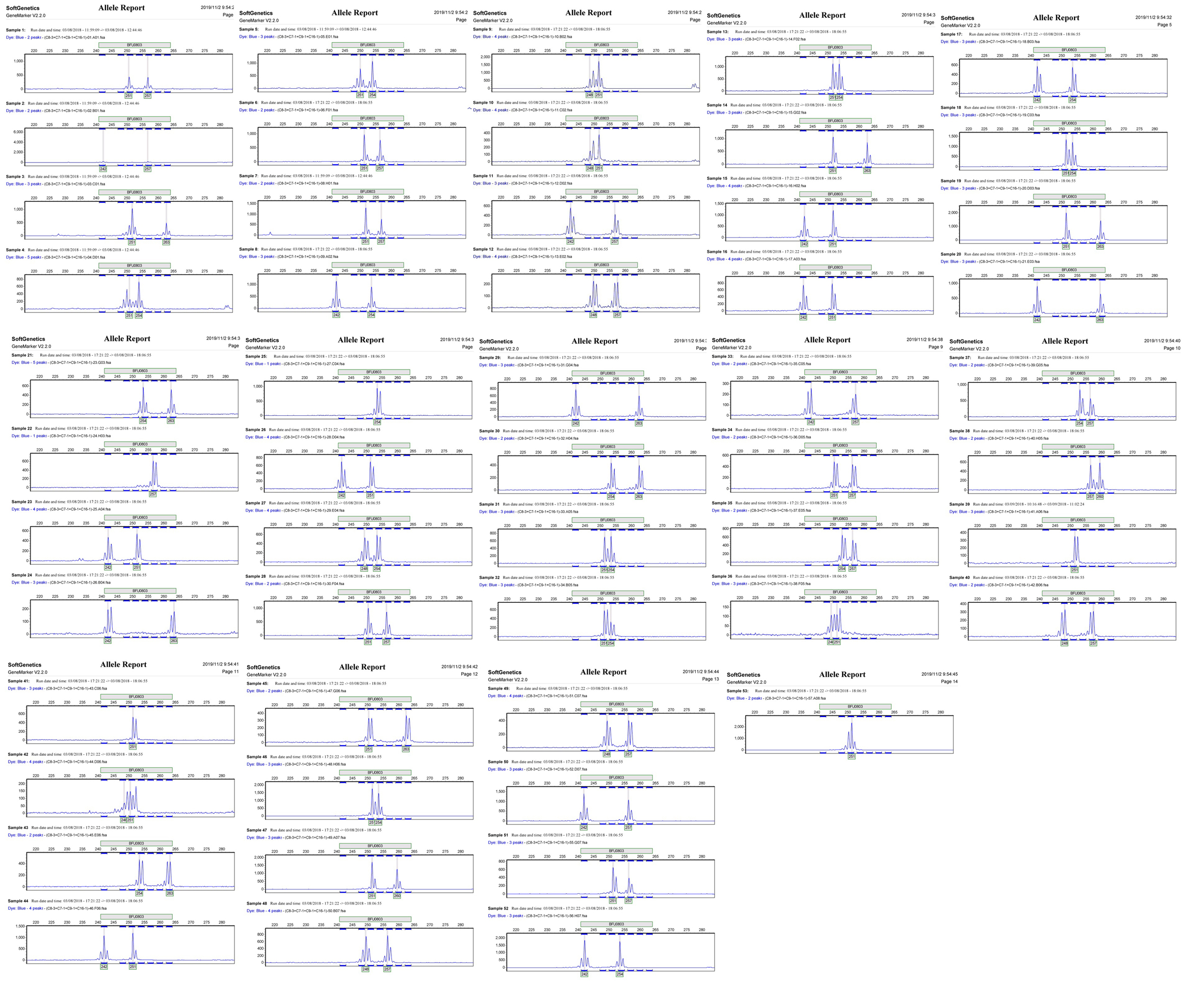

Supplement: Supplemental Information 2 [file peerj-08-8573-s006.zip › Peak maps/BFU0803.jpg]

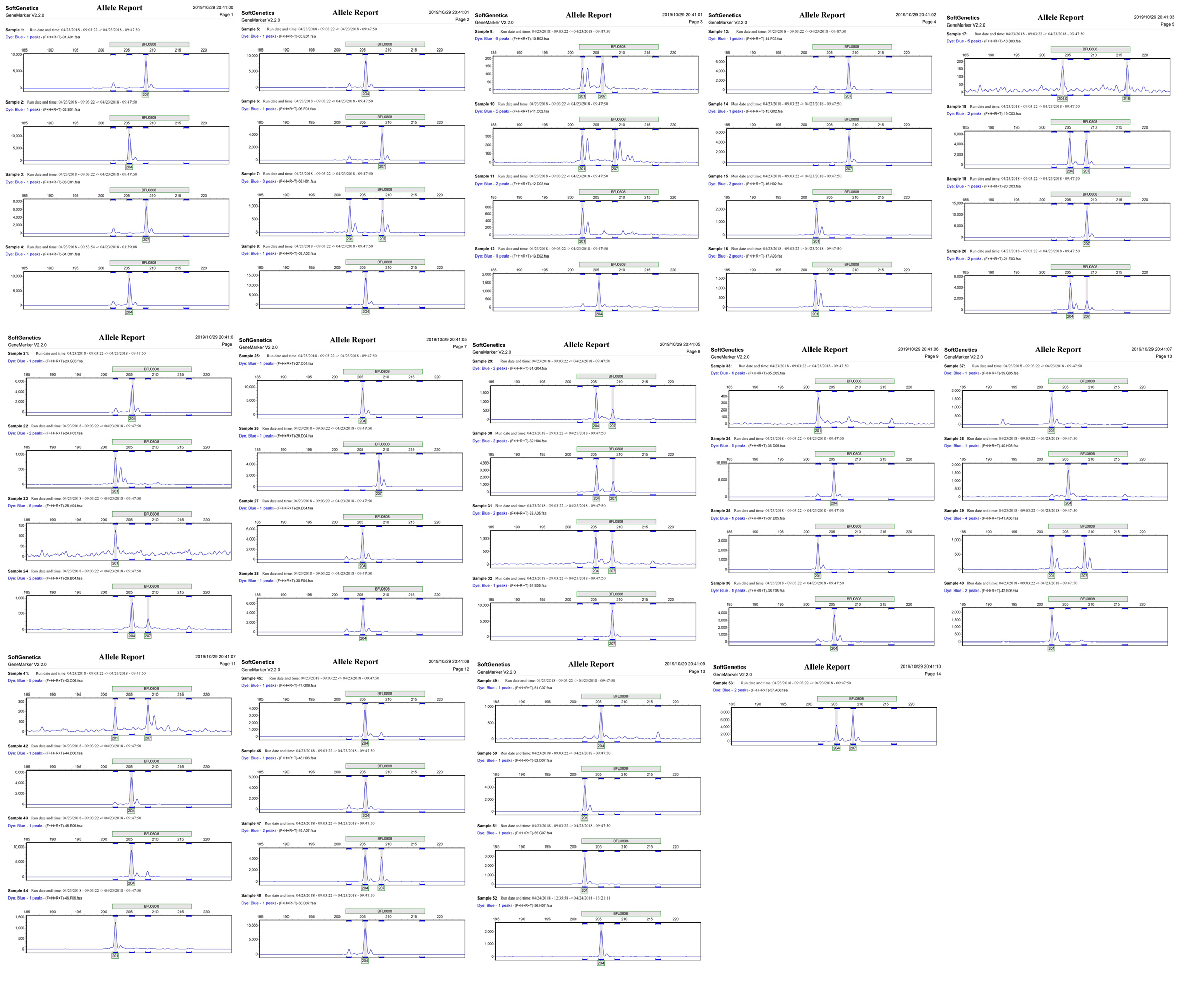

Supplement: Supplemental Information 2 [file peerj-08-8573-s006.zip › Peak maps/BFU0808.jpg]

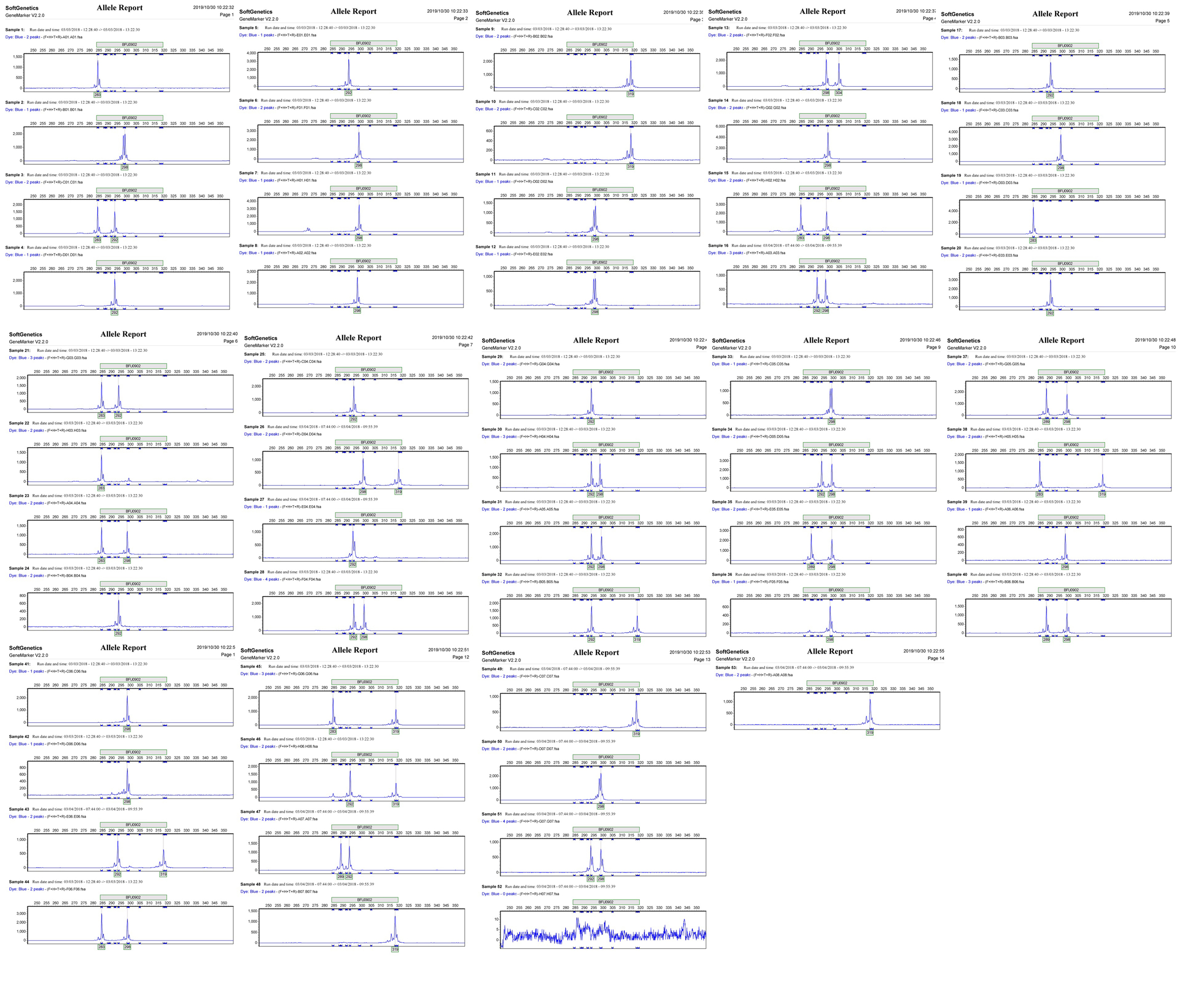

Supplement: Supplemental Information 2 [file peerj-08-8573-s006.zip › Peak maps/BFU0902.jpg]

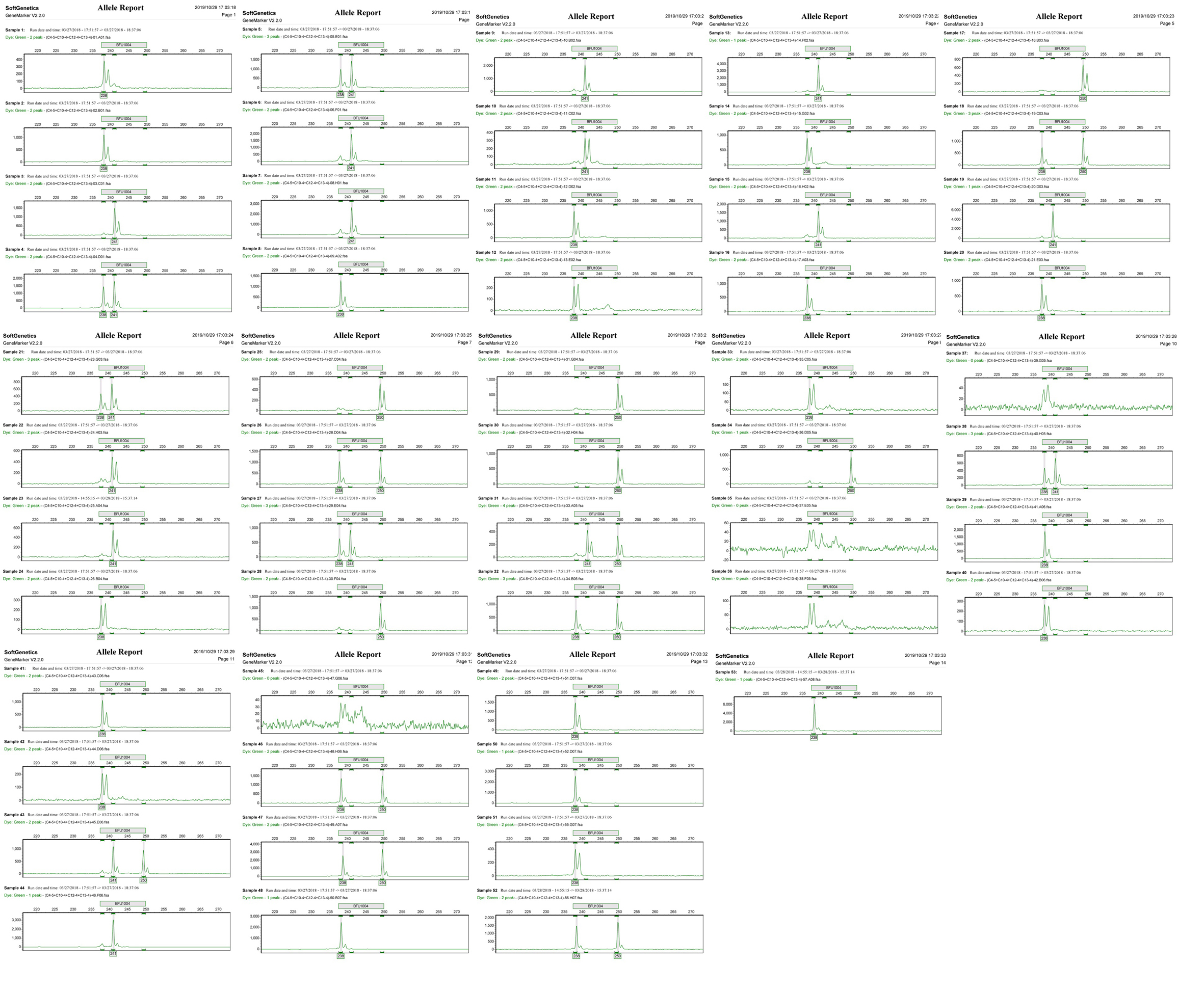

Supplement: Supplemental Information 2 [file peerj-08-8573-s006.zip › Peak maps/BFU1004.jpg]

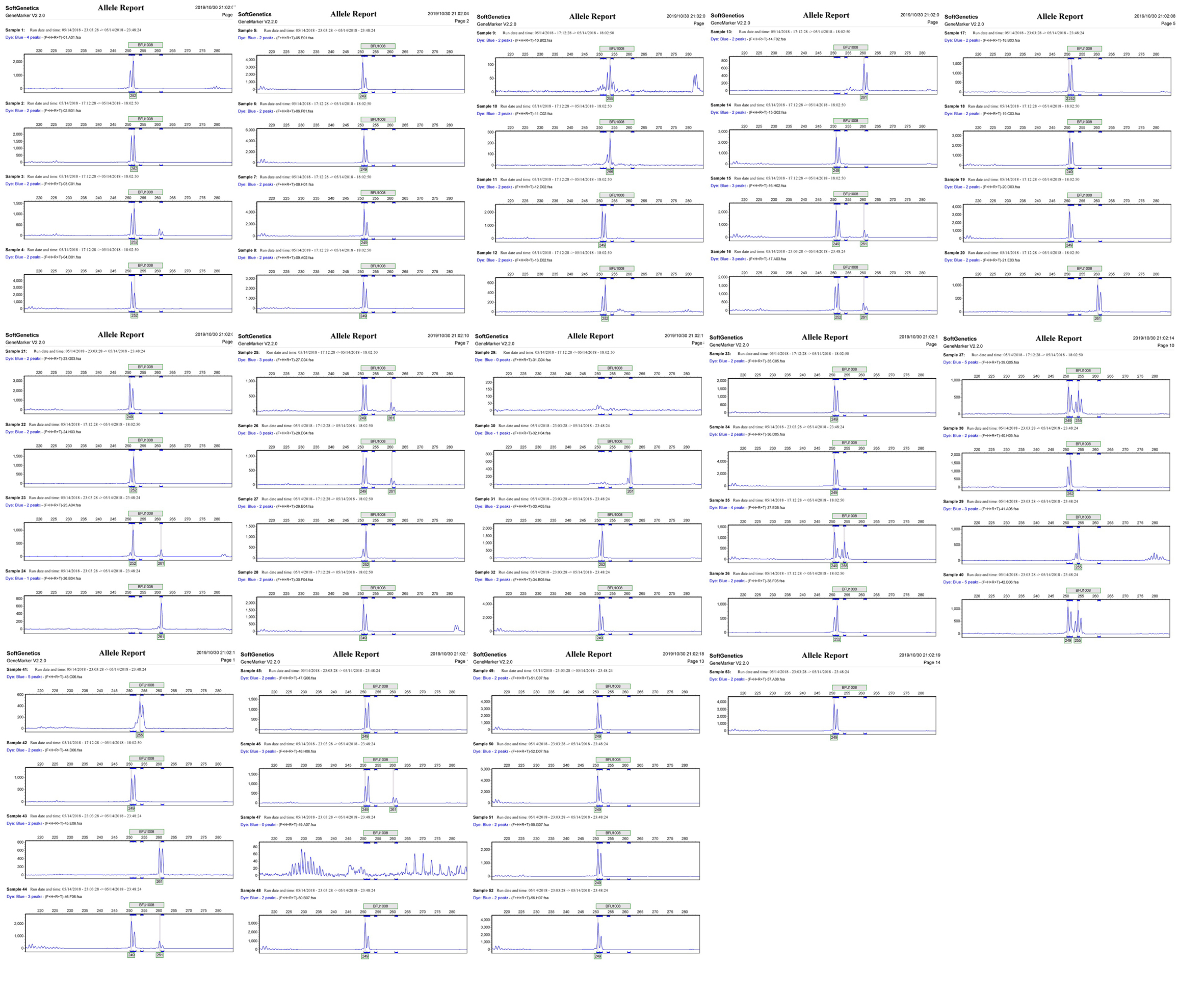

Supplement: Supplemental Information 2 [file peerj-08-8573-s006.zip › Peak maps/BFU1008.jpg]

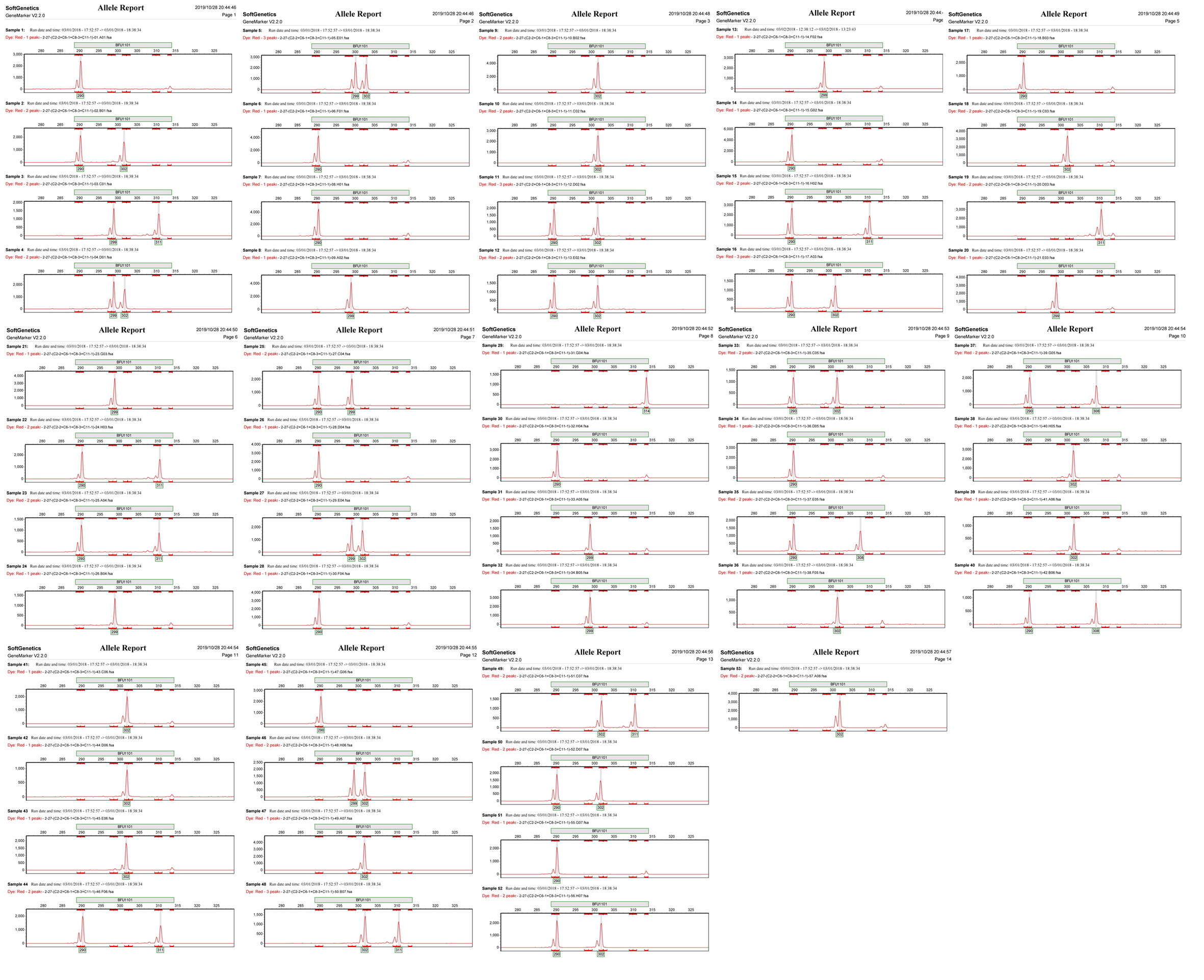

Supplement: Supplemental Information 2 [file peerj-08-8573-s006.zip › Peak maps/BFU1101.jpg]

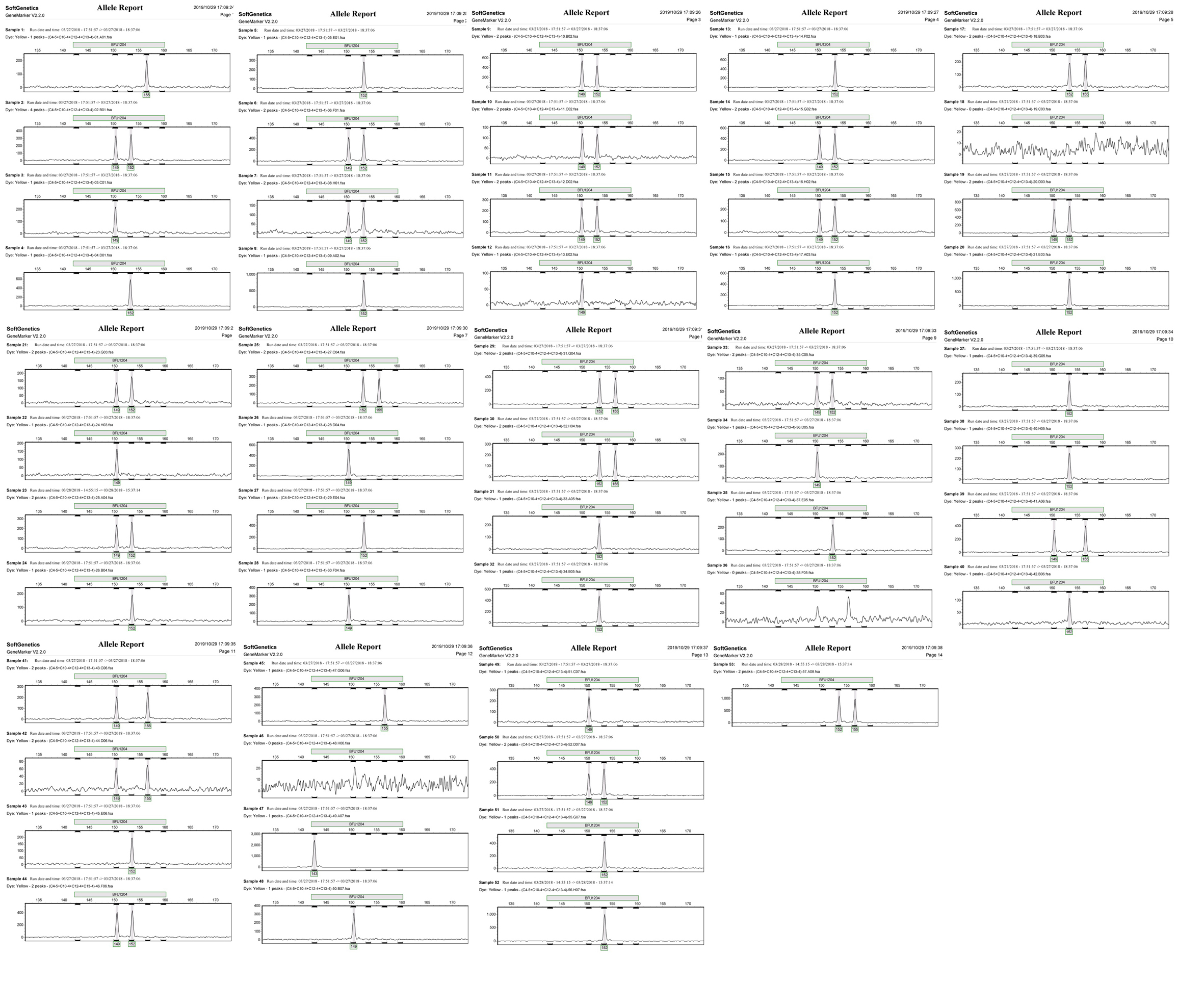

Supplement: Supplemental Information 2 [file peerj-08-8573-s006.zip › Peak maps/BFU1204.jpg]

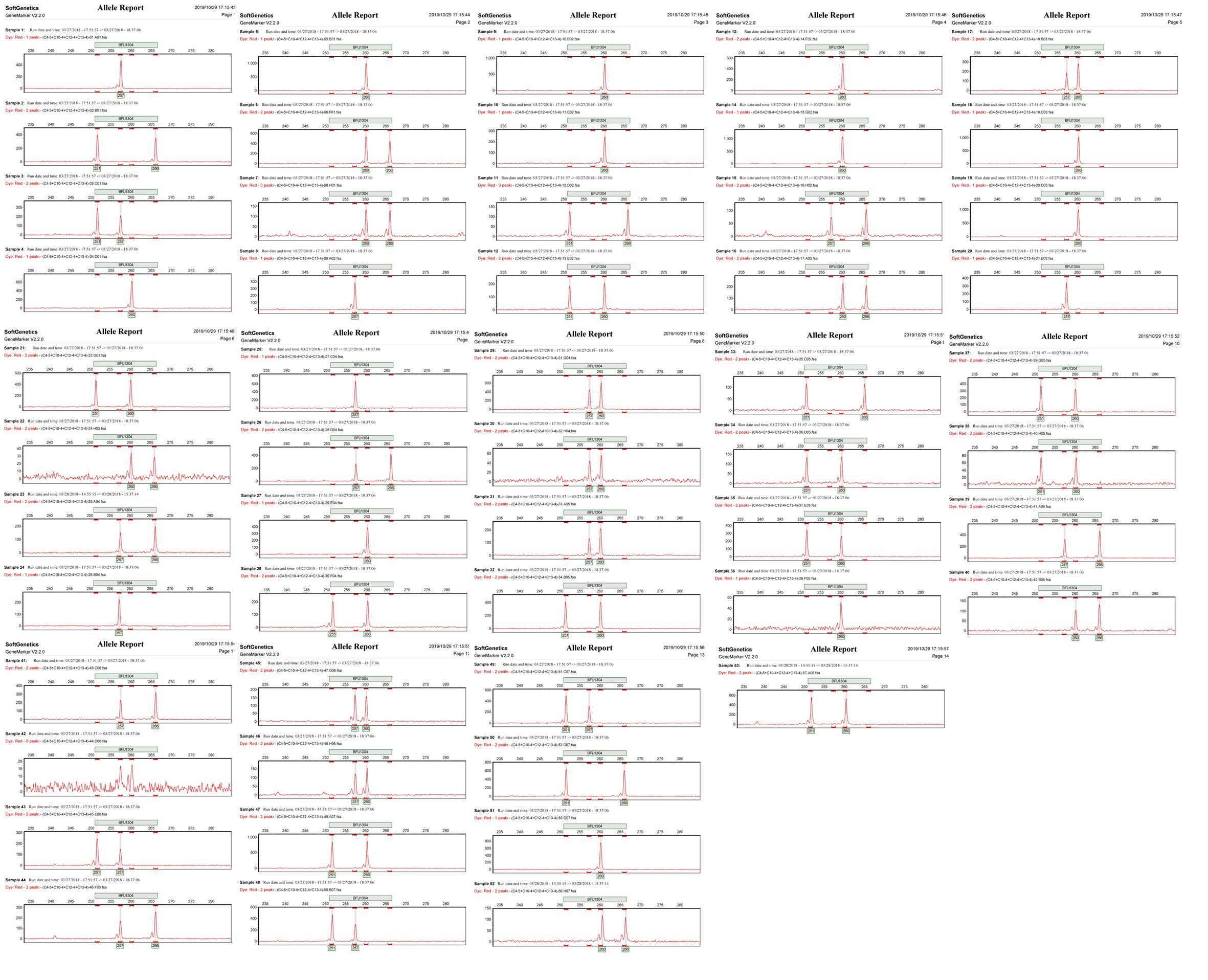

Supplement: Supplemental Information 2 [file peerj-08-8573-s006.zip › Peak maps/BFU1304.jpg]

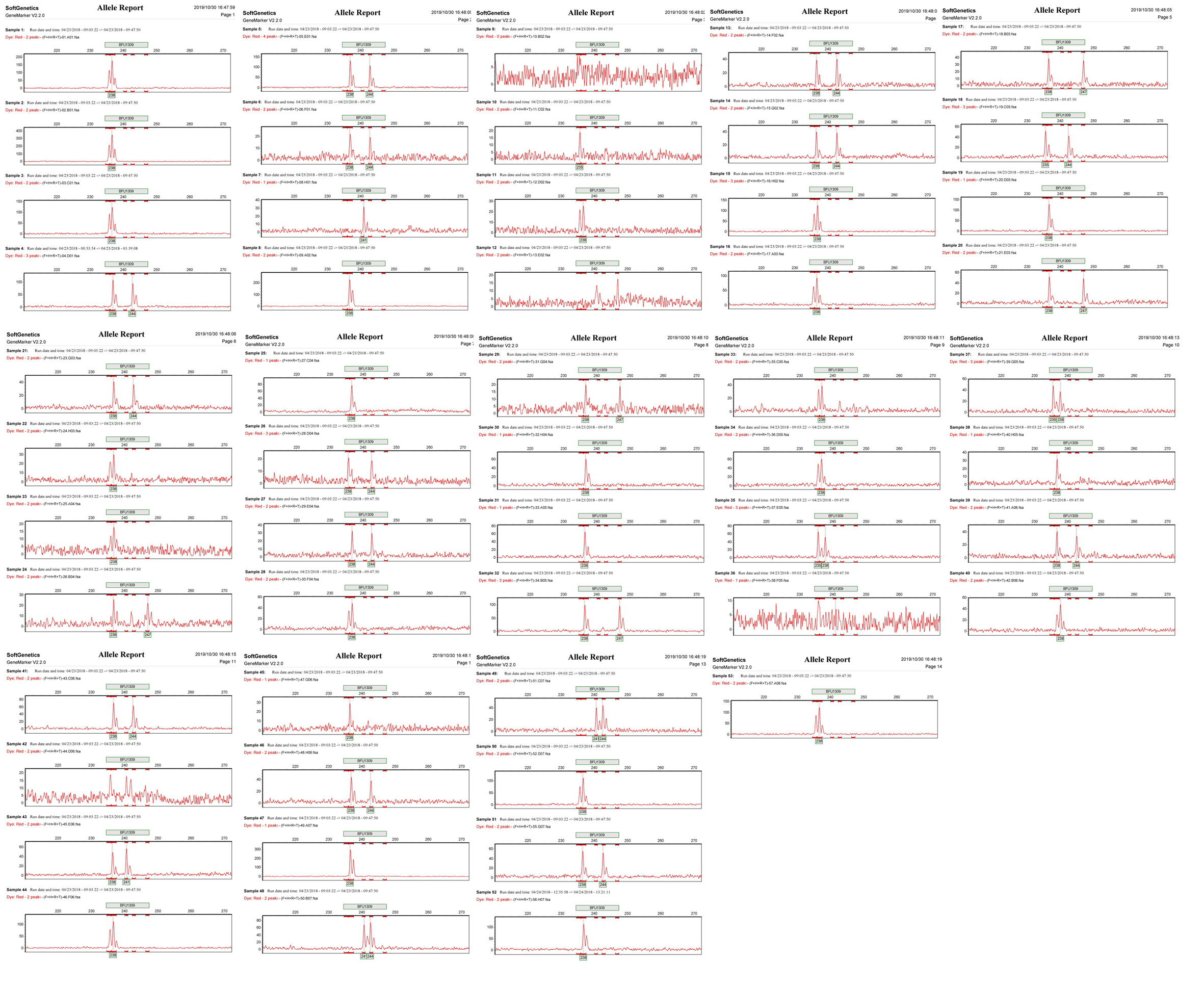

Supplement: Supplemental Information 2 [file peerj-08-8573-s006.zip › Peak maps/BFU1309.jpg]

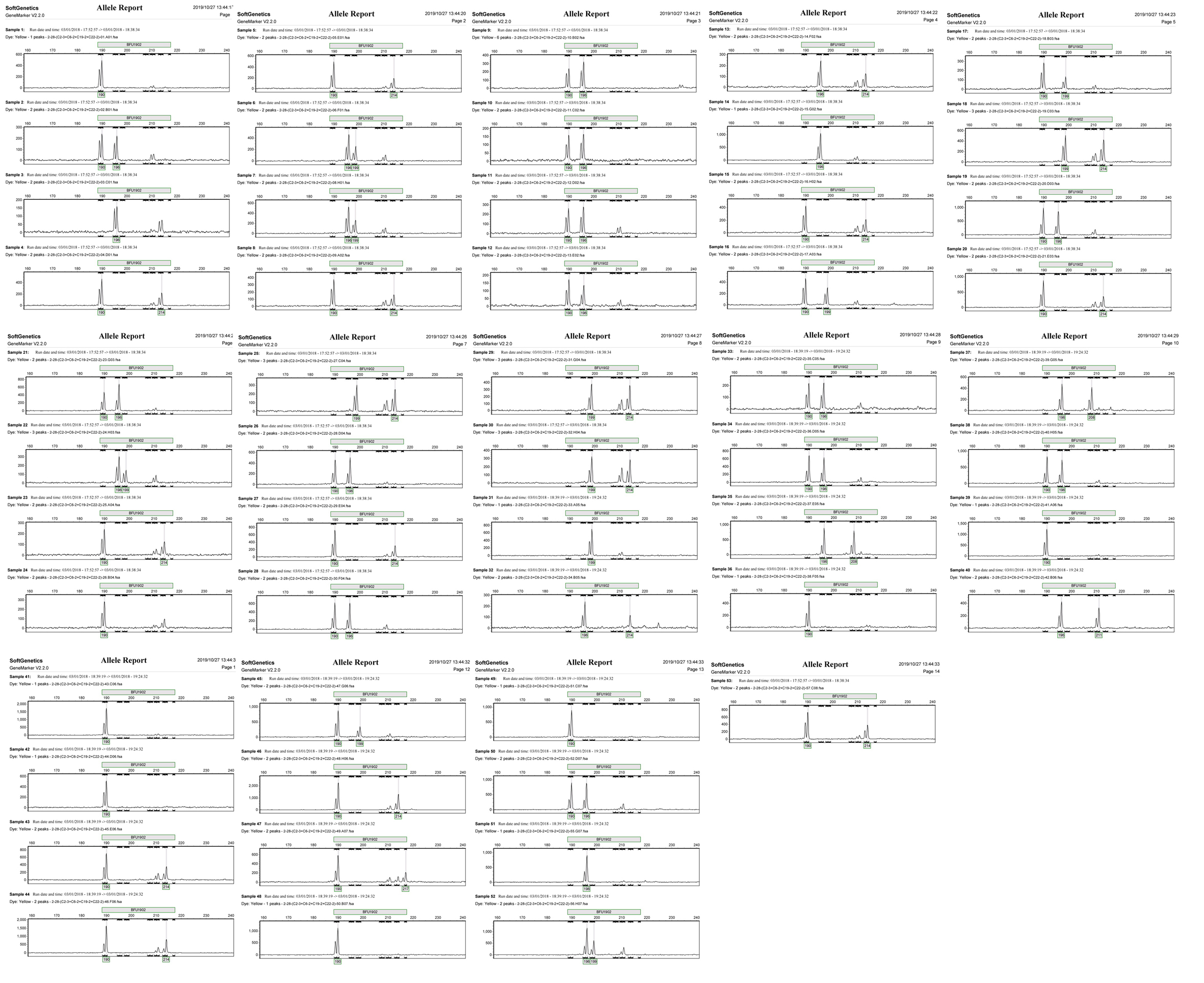

Supplement: Supplemental Information 2 [file peerj-08-8573-s006.zip › Peak maps/BFU1902.jpg]

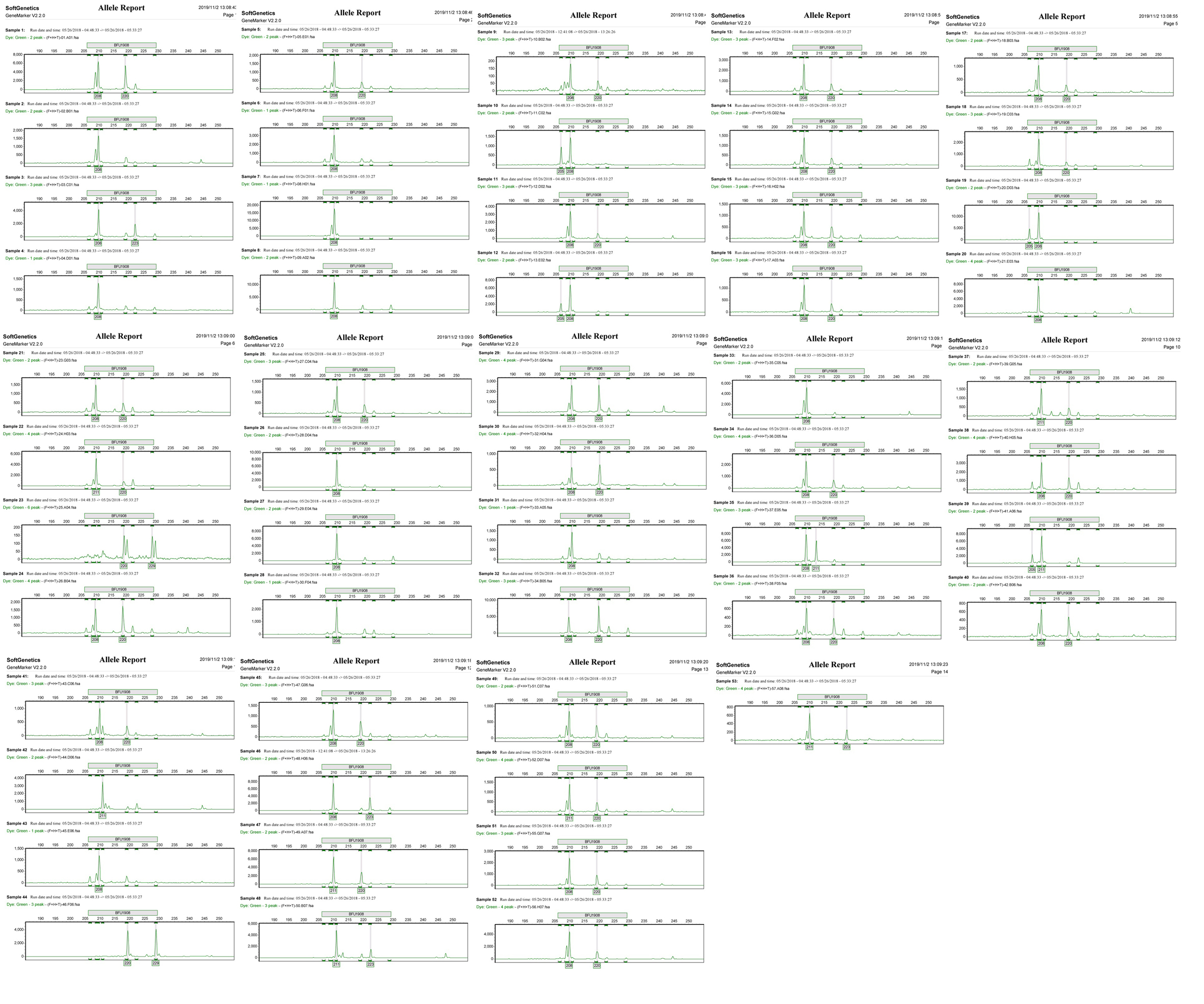

Supplement: Supplemental Information 2 [file peerj-08-8573-s006.zip › Peak maps/BFU1908.jpg]

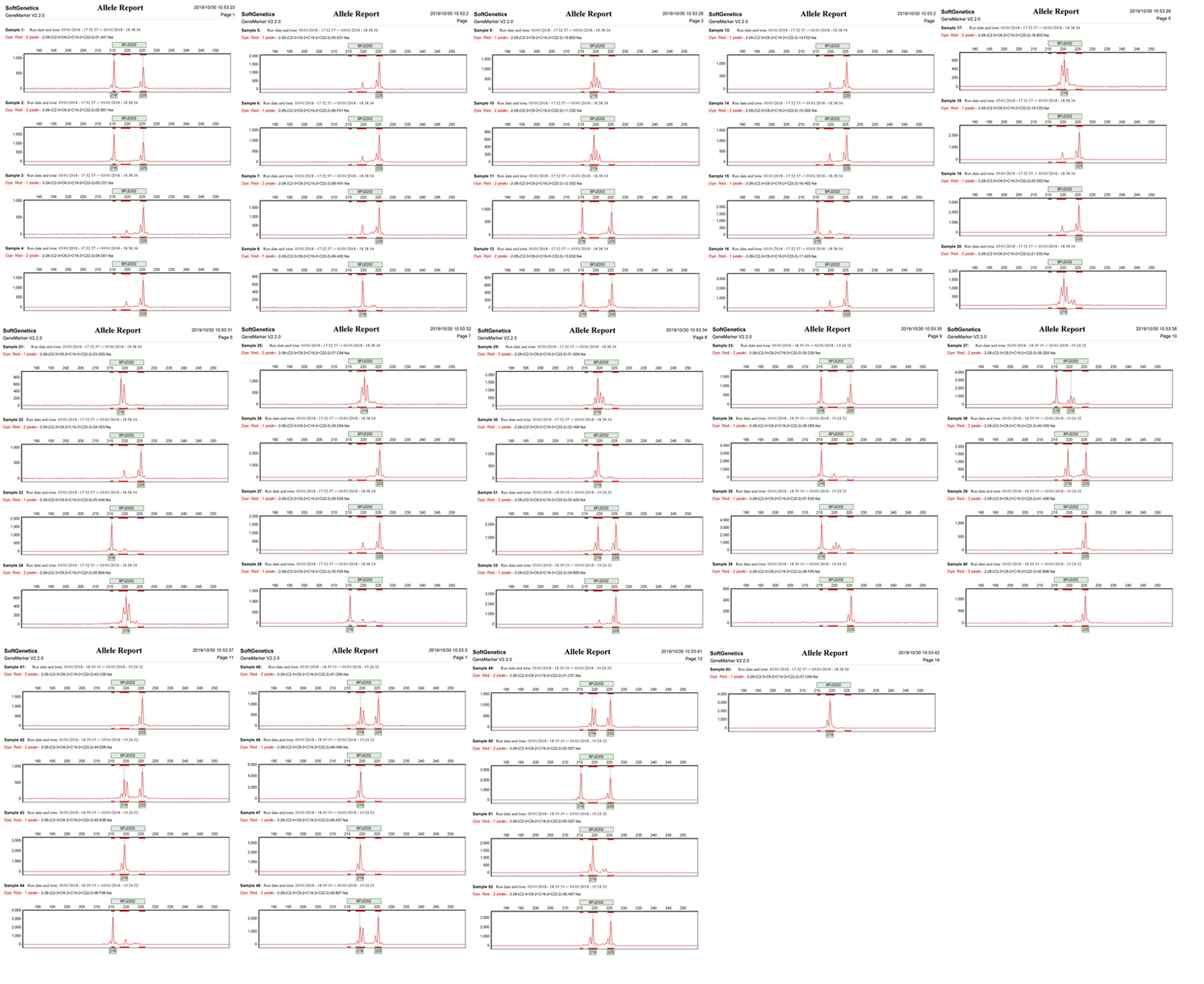

Supplement: Supplemental Information 2 [file peerj-08-8573-s006.zip › Peak maps/BFU2202.jpg]
